# Temporal phenomic predictions from unoccupied aerial systems can outperform genomic predictions

**DOI:** 10.1093/g3journal/jkac294

**Published:** 2022-11-29

**Authors:** Alper Adak, Seth C Murray, Steven L Anderson

**Affiliations:** Department of Soil and Crop Sciences, Texas A&M University, College Station, TX 77843-2474, USA; Department of Soil and Crop Sciences, Texas A&M University, College Station, TX 77843-2474, USA; Syngenta, Naples, FL, 34114, USA

**Keywords:** high-throughput phenotyping, phenomic prediction, genomic prediction

## Abstract

A major challenge of genetic improvement and selection is to accurately predict individuals with the highest fitness in a population without direct measurement. Over the last decade, genomic predictions (GP) based on genome-wide markers have become reliable and routine. Now phenotyping technologies, including unoccupied aerial systems (UAS also known as drones), can characterize individuals with a data depth comparable to genomics when used throughout growth. This study, for the first time, demonstrated that the prediction power of temporal UAS phenomic data can achieve or exceed that of genomic data. UAS data containing red–green–blue (RGB) bands over 15 growth time points and multispectral (RGB, red-edge and near infrared) bands over 12 time points were compared across 280 unique maize hybrids. Through cross-validation of untested genotypes in tested environments (CV2), temporal phenomic prediction (TPP), outperformed GP (0.80 vs 0.71); TPP and GP performed similarly in 3 other cross-validation scenarios. Genome-wide association mapping using area under temporal curves of vegetation indices (VIs) revealed 24.5% of a total of 241 discovered loci (59 loci) had associations with multiple VIs, explaining up to 51% of grain yield variation, less than GP and TPP predicted. This suggests TPP, like GP, integrates small effect loci well improving plant fitness predictions. More importantly, TPP appeared to work successfully on unrelated individuals unlike GP.

## Introduction

To improve genetic gain, plant breeders must phenotype more plants repeatedly during growth allowing higher selection intensity, accuracy, and increased statistical power (Shi *et al.* 2016; Araus *et al.* 2018; Lane and Murray 2021). High quality and quantity phenomic data are essential to develop widely applicable prediction models (e.g. phenomic predictions) to predict yield across growing environments and conditions in the near future (Bernardo 2021). To date, few phenomic data sets, approaches and applications have been reported, especially those applied in a breeding context.

Organismal fitness, such as terminal grain yield in crops, is a cumulative response of genetics (G), the environment (E), management (M), and integrated GxExM interactions temporally throughout growth. To predict cumulative fitness of an individual organism without direct measurement of that individual’s fitness, proxies such as genetic markers are used, to link measurements of relatives and predict fitness with breeding values. Traditional best linear unbiased prediction (BLUP) derived breeding values (Henderson 1975) were modified by Bernardo (1994) where genotypic marker data of parental inbreds was combined with the yield data of the related single cross hybrids to predict yield performance of the single cross hybrids, known as genomic BLUP (GBLUP). However, prediction accuracies dropped dramatically when yield of unknown (previously untested) parental lines-derived hybrids was predicted (Bernardo 1996a,b). Various genomic-based statistical models have been developed after the traditional GBLUP approach with advent of genomic technology (Whittaker *et al.* 2000; Meuwissen *et al.* 2001; Endelman 2011). These methods have been applied extensively as genome-wide marker facilitated selection also known as genomic selection in plants (Bernardo and Yu 2007). Predicting the performance of previously untested genotypes in both tested and untested environments remains the central problem in plant breeding selections, and new approaches to address this challenge are needed. Genomic selection to estimate genotype fitness, as measured by terminal grain yield, relies on manually collected phenotype data which is resource intensive to collect. Phenotypic characteristics of cumulative complex traits are often not accurately predicted in genomic selection (GS) because of (1) the different interplays of genes on phenotype throughout different growth stages, (2) different effect sizes of the same genetic markers on phenotype of complex traits at different growth stages, and (3) different sources of phenotypic variation of the complex traits at different growth stages (Wu *et al.* 2004; Bac-Molenaar *et al.* 2015; Campbell *et al.* 2017; Feldman *et al.* 2017; Anderson *et al.* 2019; Ward *et al.* 2019; Adak, Conrad *et al.* 2021; Adak, Murray, Anderson *et al* 2021; Adak, Murray, Božinović *et al.* 2021). Tools that can inexpensively evaluate individuals throughout growth, as they interact with their environment, would therefore be a valuable addition to predicting an organism’s fitness. Unoccupied aerial systems (UAS) are now able to provide these insights, frequently evaluating individuals temporally throughout growth. However, to date, fitness predictions from UAS alone have not been compared to the standard method of genomic prediction (GP). In applied breeding programs, GP and temporal phenomic prediction (TPP) both have benefits but are not interchangeable. Both genomic and phenomic prediction can decrease labor, time and resources, per new elite variety developed. In the training of prediction models, both require measurements (DNA for genomics, remote sensing for phenomics) and a dependent variable, such as yield, measured across relevant germplasm and environments. After a prediction model has been built, application differed. GP is most valuable when seed is genotyped before planting, reducing plot number and cost to maintain and harvest the crop. Genotyping on each non-segregating inbred or hybrid entry only needs to be done once. Genotyping before planting works in industry breeding programs where dedicated seed DNA extraction and genotyping pipeline resources exist but is challenging in the public sector where such infrastructure is rare. If GP results are obtained after the seed is planted, it is still beneficial for breeder selection, but does not substantially decrease costs. Also, in general, the measured dependent trait (e.g. yield data by combine) is likely more reliable for the genotypes behavior in that environment (Bernardo 2021). GP can estimate the G component well but largely cannot predict GxE or E in untested environments without additional information such as weather data or known similarity between environments, as well as crop modeling (Jarquín *et al.* 2014; Jarquin *et al.* 2021; Rogers and Holland 2021). Undertaking GP is valuable also for the ancillary genomic data useful for determining relatedness, genetic architecture of traits or making genetic associations and mapping, all publishable topics, but may not be helpful in breeding.

Phenomic prediction will play a different role in breeding programs. It does not immediately decrease the number of plots planted or plots maintained for most of the season, but these plots need not be harvested. Harvesting can be the most expensive, labor intensive and dangerous procedure in yield trials, especially moving harvest equipment to different locations. Because of this streamlining and miniscule marginal costs of additional plots, phenomic prediction may result in breeders increasing the number of plots or locations, if they need not be harvested, which will increase genetic gain. Phenomic prediction may also make it possible to decrease plot size since remote sensing prediction can likely be done on a fewer plants. TPP is most valuable for identifying superior plots early and throughout the growing season; so far, evidence suggests the most predictive time for grain yield may be before flowering (Adak, Murray, Božinović *et al.* 2021). This early indication of superior performance allows faster cycling to off-season nurseries for recombining and advancement, or at least more preparation time for an off-season nurseries seed. Uniquely, phenomic prediction gets at not only G, but GxE and E in measurements—as a simple example, if healthy and vigorous plants with good fitness across the entire location can indicate E. Then both G and the repeatable component of GxE can be observed as the differences between individual genotypes deviating from location mean. Because of the integration of G, E, and some GxE, there is already indication from near-infrared spectroscopy (NIRS) grain phenomic predictions that models are predictive across diverse germplasm and environments (Lane *et al.* 2020), which is uncommon for GS. There are ancillary benefits of TPP data as well. Temporal data can help understand how different varieties interact with the environment in real time to discover important growth stages and conditions, along with genetic interactions that impact yield. This could allow deliberate pyramiding of genetics showing elite performance at different growth stages—unlike yield and GS alone which are cumulative measures. Additionally, temporal data can predict stress before loss occurs which can increase the quality of yield data (DeSalvio *et al.* 2022). Ultimately, identifying major causal loci underlying phenomic predictions success for complex traits can be useful to understand the underlying biology of organismal fitness over growth. Finally, because temporal phenomic data are relatively new, when compared with over 30 years of genomic data, it is likely unanticipated advantages and disadvantages will continue to be discovered.

To evaluate fitness prediction of UAS-based phenomics tools, the genotypic value of each hybrid must be produced, these can be estimated from vegetative indices (VIs) and structural measurements (canopy height) collected temporally throughout growth. VIs at a single or few time points have been shown to be highly predictive of plant health, phenology and yield (Araus and Cairns 2014; Rutkoski *et al.* 2016; Adak, Murray, Božinović *et al.* 2021). With temporal flights, these are now being collected over a fourth dimension, time, where interactions with the environment can be observed as they occur. Correlations between temporal VIs with yield and flowering times, as well as machine learning models can be used to investigate predictive abilities for fitness traits (yield and flowering times). Phenomic predictions made from temporal VIs and canopy height can be compared with traditional GPs. Ultimately, identifying major causal loci underlying phenomic predictions success for complex traits can be useful to understand the underlying biology of organismal fitness over growth. Here we report phenomic data-driven selection for complex traits in maize breeding. We conducted UAS surveys with multispectral and red–green–blue (RGB) sensors to collect image-based temporal predictors throughout maize growth stages. We compared phenomic-based prediction accuracy to that of GP, explored temporal shifts in image-based phenotypic variation explained by genome-wide markers, and conducted association mapping utilizing temporal image-based phenotypes to identify biologically important loci.

## Materials and methods

Using the Genome to Fields initiative’s 2017 germplasm, 280 unique maize hybrids were grown under optimal management (OM) and 230 were grown under stressed management (SM, no irrigation, low fertilizer) near College Station, Texas. Two replications were used in a randomized complete block design with each hybrid grown as 2 consecutive row plots. The hybrids were primarily Stiff Stalk inbred lines crossed with Non Stiff Stalk inbred lines, mostly derived from expired plant variety protected lines, although hybrids derived from elite exotic germplasm and recombinant inbred lines as well as commercial checks were also included (https://doi.org/10.25739/w560-2114; accessed 2022 November 11, McFarland *et al.* 2020). The goal of this germplasm was to provide breadth as well as depth for some more targeted questions. While many other hybrids were created to be included, sufficient seed supply was a primary consideration for determining the specific hybrids planted.

### UAS surveys and image processing

A Phantom 3 Professional rotary-wing UAS, equipped with a 12-megapixel RGB DJI FC300X camera, flown 25 m above the ground (TPP_RGB) for 16 flights. Additionally, a Tuffwing UAS equipped with a MicaSense RedEdge-MX multispectral camera was flown 120 m above the ground (TPP_Multi) for 12 flights. Images were collected with 80% forward and side overlap in both surveys. Raw images were processed in Agisoft Metaphase Professional software to generate the 3D point clouds and orthomosaics (Supplementary Table 1 in File 1) (Murray *et al.* 2019).

### Phenomic data extraction pipeline

Environmental Systems Research Institute, Inc. (ESRI) shape file were constructed using R/UAStools::plotshpcreate function (Anderson and Murray 2020) and applied to each survey’s respective orthomosaic (.tif files) and 3D point clouds (.las or .laz files) to extract plot level image-based phenotypes. VIs (Supplementary Table 2 in File 1) for each flight date (from 27 days after planting until 144 days after planting) were extracted using the *FIELDImageR* package (Matias *et al.* 2020) for each UAS survey (Supplementary Materials and Methods in File 1). Plot-based 99th percentile temporal plant heights (canopy height measurement; CHM) were extracted from 3D point clouds following the methods of (Anderson *et al.* 2019) (Supplementary Materials and Methods in File 1) for each UAS survey.

### Experimental design and nested model for phenomic data

To analyze the temporal VIs and CHM, a custom nested design was applied to raw data of each VI and CHM belonging to each row plot in OM and SM, where experimental design and maize hybrids were treated as nested within drone flight times (Supplementary Materials and Methods in File 1). Hybrids nested within flights results were used to predict 4 agronomically important traits (grain yield: GY, days to anthesis: DTA, days to silking: DTS, and terminal plant height: PHT) within and between the trials. GY (t/ha) was obtained at the end of season for each plot using a plot combine and adjusting moisture to 15.5%; DTA and DTS were recorded when anthesis and silking of at least 50% of each plot emerged; PHT was measured manually at the end of season one time for each plot in that 5 plants were measured within each plot. Correlation in Supplementary Figs. 5, 6 and 9 were calculated using genotypic values of hybrids predicted by Supplementary Equation (2).

### Machine learning-based phenomic prediction models

Manually collect phenotypes (GY, DTA, DTS, and PHT) were predicted from UAS measures using linear, elastic net, ridge, lasso, and random forest regressions. For all results in this study, TPP_RGB and TPP_Multi contains the VIs at all time points belonging to each hybrid that were obtained from UAV images captured by RGB and multispectral camera respectively (Supplementary Dataset 1). Prediction models were trained using a random sampling of 70% of the common maize hybrids (tested genotypes). The remaining 30% were used as the validation dataset (untested genotypes). Models were trained using tested genotypes grown in OM trial (tested environment) while the SM trial served as the untested environment. Four cross-validation schemes (CVs) were conducted as follows: (1) tested genotypes in tested environment (CV1), (2) untested genotypes in tested environment (CV2), (3) tested genotypes in untested environment (CV3), and (4) untested genotypes in untested environment (CV4) (Li *et al.* 2018). CV1 was used as negative control to show the overfitting while CV2, CV3, and CV4 were used as merits of prediction accuracies in temporal phenomic and GP. Additional details on phenomic prediction models and prediction steps are available in Supplementary Materials and Methods in File 1. Prediction accuracies were compared based on mean numeric values from 500 iterations of each CV; because the same iterations were used for different methods, they are directly comparable.

### Genomic prediction for phenomic data

Genome-wide prediction was applied to 540 image-based phenotypes (35 VIs and CHM belonging to up to 16 flight times) of the 158 maize hybrids in TPP_RGB of OM using 153,252 SNPs, temporal GP model was explained in Supplementary Materials and Methods in File 1.

### Phenomic prediction vs genomic prediction

GBS marker data for GP and 2 sets of phenomic data (TPP_RGB and TPP_Multi) were used to conduct GP and phenomic prediction for maize grain yield (GY). A total of 118 G2F (Genomes to fields; https://www.genomes2fields.org/) maize hybrids were used to compare the predictive ability between the genomic and phenomic data sets. Four cross-validation schemes were applied as explained in “*Machine learning-based phenomic prediction models*” section. Additional details regarding phenomic prediction vs GP are available in Supplementary Materials and Methods in File 1.

### Association mapping for phenomic data

**Fig. 1.**
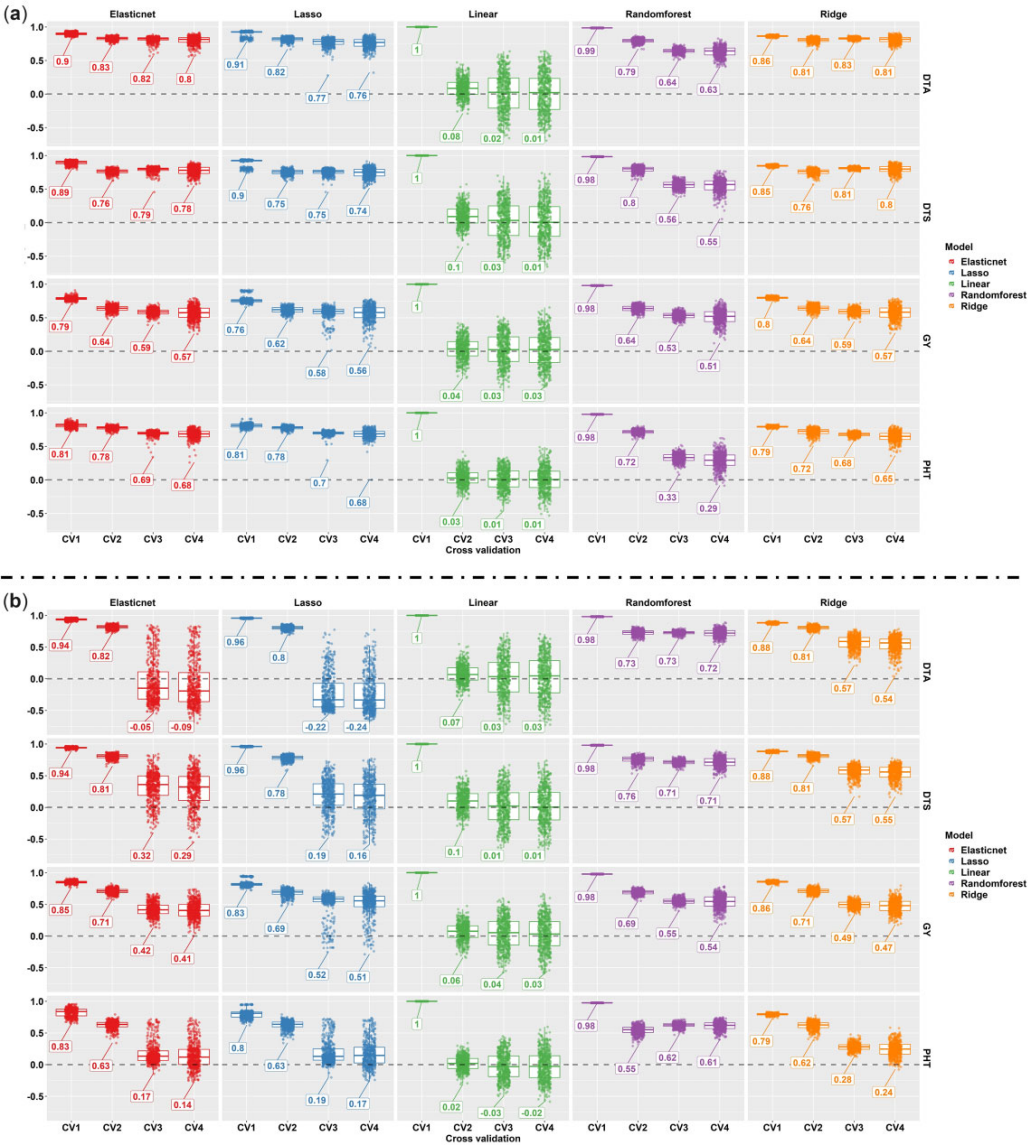
Prediction accuracy (on the *y* axis) of the phenomic prediction obtained by each model for 4 cross-validation schemes (on the *x* axis) belonging to each predicted variable (from left to right) in phenomic prediction. a) The prediction performance of TPP_RGB phenomic data derived from HTP platform including 25-meters elevation with RGB sensor. b) The prediction performance of TPP_Multi phenomic data derived from HTP platform including 120-m elevation with multispectral sensor. The boxes in each point cloud show mean values of prediction accuracies.

The image-based VIs and Weibull_CHM (temporal plant height fit based on Supplementary equation 1 in File 1) were converted to cumulative area under the curve (AUC) values and used as trait data in a genome-wide association study (GWAS) (Supplementary Materials and Methods in File 1). Association mapping was conducted using 158 maize hybrids and 101,100 genotyping by sequencing (GBS) SNP markers, implementing 3 multiple loci test methods; (1) fixed and random model circulating probability unification (FarmCPU) (Liu *et al.* 2016), (2) multiple loci mixed model (MLMM) (Segura *et al.* 2012), and (3) bayesian-information and linkage-disequilibrium iteratively nested keyway (BLINK) (Huang *et al.* 2019) (Supplementary File 1 in Materials and Methods). Linkage disequilibrium (LD) estimates were used to identify candidate genes within LD blocks ($R^{2}$ ≥ 0.8) of colocalized SNPs (Supplementary File 1 in Fig. 1).

## Results

### Variance decomposition and repeatability estimates demonstrate UAS sensor-based phenotypes were genetically stable

Variance component decomposition of the 83 sensor-based VIs (35 RGB and 54 multispectral) demonstrated UAS sensor-based data were statistically repeatable and biologically meaningful with a genetic basis. The rotary-wing equipped with an RGB (3 band, 12 MP) sensor flown at 25 m resulted in ∼1 cm pix^−1^ image resolution and had higher repeatability than the Tuffwing platform equipped with a multispectral (5 band, 3.8 MP) sensor flown at 120 m (∼8 cm pix^−1^). The main source of phenotypic variation for both platforms was explained by the temporal flight component ($\beta_{i}$ component in Supplementary Equation 2 in File 1) of the nested design (31–96%) showing a temporal plasticity of maize spectral reflectance signatures throughout the plants growth cycle (Supplementary Figs. 2 and 3 in File 1). Genetic variance ($\Omega_{i(j)}$ component in Supplementary Equation 2) was slightly greater for the higher resolution-low altitude RGB Phantom 3 (1.5–5.2%; Temporal repeatability (TR): 0.46–0.77) phenotypes compared to the lower resolution-high altitude RGB Tuffwing (1.1–4.5%; TR: 0.26–0.66) and lower resolution-high altitude multispectral Tuffwing (0.5–3.4%; TR: 0.28–0.62) phenotypes (Supplementary Figs. 2 and 3 in File 1). The repeatability estimates over the 35 RGB phenotypes were highly correlated (*r* = 0.71) between the 2 sensor systems, although repeatability was improved by 0.08 on average, when implementing the higher resolution-low altitude RGB platform. Noticeable improvements in repeatability estimates (>0.1) were achieved for 13 RGB VIs and 6 VIs repeatability were reduced (<0.06) when implementing the higher resolution-low altitude RGB platform (Supplementary Figs. 2 and 3 in File 1). Overall, significant genetic variation was attributed to all VIs on both platforms, useful in predictive modeling of important agronomic traits. Relevantly, the largest proportion of explained % variation for important agronomic traits flowering times (DTA, DTS), grain yield (GY), and terminal plant height (PHT) with repeatability of ∼0.9 for flowering times and yield, 0.8 for PHT (Supplementary Fig. 4 in File 1).

### Temporal correlation

Temporal correlations between temporal genotypic values of VIs in TPP_RGB and GY showed that 14 of 35 VIs achieved a correlation above 0.50 (up to 0.61) (Supplementary Fig. 5 in File 1). However, temporal correlation between temporal genotypic values of Vis in TPP_Multi and GY showed that 14 VIs (calculated using only red, green blue bands) and 40 VIs (calculated using red, green blue, red edge, and NIR bands) achieved correlations above 0.50 (up to 0.70) (Supplementary Fig. 6 in File 1). Correlation between sensor-based VIs and GY varied depending on the flight dates. High correlations were found between VIs belonging to certain time points in both TP_RGB and TPP_Multi and GY demonstrating that temporal VIs tend to synchronize with GY in maize hybrids indicating potential measures for predicting yield.

### Phenomic prediction using high dimensional UAS data

Temporal genotypic values of VIs for each genotype at each timepoint in TPP_RGB and TPP_Multi followed unique trajectories (Supplementary Figs. 7 and Fig. 8 in File 1) visually discriminating low, mid, and high yielding maize hybrids. Phenotype data of VIs at different time points had different discriminative ability for yield. This led us to test the predictive ability of 2 sets of phenomic data derived from different sensors and resolutions utilizing the different prediction models. To assess the multicollinearity of each phenomic data measure, correlation coefficients were calculated. Correlation results of each phenomic data showed that correlations were fluctuating between −1 and 1, and VI’s were found to be less correlated at different time points (Supplementary Fig. 9 in File 1).

The 3 machine learning models had high prediction accuracy (>90%) for all 4 agronomic traits (GY, DTA, DTS, and PHT), especially when compared with the linear model when temporal phenotypes in TPP_RGB (Fig. 1a) and TPP_Multi (Fig. 1b) phenomic data of 280 hybrids were used as predictors. The linear models had the highest prediction errors (RMSE; root mean square error and MAE; mean absolute error) and lowest *R*^2^ (Supplementary Fig. 10 in File 1). Penalized linear regression methods (Ridge, Lasso, and Elasticnet) generally performed similar or better than non-linear models (Randomforest) for predicting DTA, DTS, GY, and PHT when TPP_RGB phenomic data were used in prediction performances for untested genotypes in tested environment (CV2), tested genotypes in untested environment (CV3), and untested genotypes in untested environment (CV4) (Supplementary Fig. 1 in File 1). Using penalized linear regression models (ridge, also and elastic net) with TPP_RGB predicted the GY, DTA, DTS, and PHT greater than TPP_Multi in untested environment-related prediction scenarios (CV3 and CV4); however, TPP_Multi predicted the GY greater than TPP_RGB in tested environment-related prediction scenario (CV2) (Fig. 1). Among the penalized linear regression methods, ridge regression achieved the greatest prediction accuracy. For instance, prediction accuracy of ridge regression was highest compared to lasso and elastic net in prediction the GY in CV2 using TPP_Multi; its prediction accuracy also highest compared to lasso and elastic net in CV3 and CV4 using TPP_RGB. Prediction accuracies of the prediction models for DTA, DTS and PHT in CV1 to CV4 were given in Fig. 1. These results demonstrate that the reduction in resolution, increased spectral bands, and increased sensor cost of incorporating the multispectral bands did not significantly improve model performance in CV3 and CV4 schemes.

### Variable importance scores of the machine learning models

To understand potential biological causes behind the most accurate predictions, variable importance scores were derived from the prediction models to identify critical predictor/time point combinations for TPP_RGB and TPP_Multi phenomic data sets (Supplementary Figs. 11 and 12 in File 1). Different contributions of VIs and Weibull_CHM at multiple time points were important among both phenomic datasets in the prediction of GY, DTA, DTS, and PHT (Supplementary Figs. 11 and 12 in File 1). For instance, the TPP_RGB red chromatic coordinate index (RCC) and TPP_Multi modified nonlinear index values (MNLI) belonging to various time points, either before or after flowering times, for all predicted variables were identified by all machine learning models consistently and are therefore critical VI/timepoints combinations for all predicted variables (Supplementary Figs. 11 and 12 in File 1). This demonstrates an ability of machine learning models to identify important image-based phenotypes for future UAS surveying efforts and provides foundational insight toward understanding the biological importance of images-based phenotypes within a plant’s growth cycle.

### Genome-wide association mapping results

To gain further insight into biological significance of successful predictions, GWAS peaks were identified using area under curve values (Supplementary Figs. 13 and 14 in File 1) of each high resolution VI and Weibull_CHM in the TPP_RGB phenomic data set of 158 hybrids (Supplementary Figs. 13 and 14 in File 1). Area under curve was presented as a summation of all timepoints, rather than each timepoint individually to keep the results interpretable and robust. Still, a total of 241 GWAS peaks were identified across the 36-temporal image-based phenotypes in TPP_RGB. Five genomic regions had significant loci for VIs and candidate genes of relevant interest (Supplementary Results in File 1). Two genomic regions were identified as hotspots (the fourth bin in chr2 and eighth bin in chr4) having GWAS peaks belonging to 24 VIs discovered across all 3 tested GWAS models (Supplementary Fig. 14 and Dataset 2 in File 1). A 15 kb genomic distance around the GWAS peaks was scanned to determine candidate genes based on the calculated LD decay (Supplementary Fig. 15 in File 1). LD patterns of both hotspots were visualized along with 6 candidate genes with functions described in Supplementary Fig. 15 in File 1.

A hotspot was identified at 36,828,844 bp on chromosome 2 (*chr2_1*), identified by the excessive red, modified green red, normalized difference, Normalized green red difference, and visible atmospherically resistant indices by the 3 GWAS models consistently explaining 8–13% phenotypic variation (Supplementary Dataset 2). The *chr2_1* peak is inside *GRMZM2G023204* (chr2:36827859.36,829,876; B73 RefGen_v4), a putative protein kinase domain that catalyzes the function of protein kinases. Another candidate gene (∼4kb away from *chr2_1*) is *GRMZM2G021560* (*pebp25*; chr2:36,779,809.36,782,444; B73 RefGen_v4) a member of phosphatidylethanolamine-binding proteins (PEBPs) that regulate floral transitions (Danilevskaya *et al.* 2008) as well as that *GRMZM2G021560* found to be expressed at the early vegetative stage (e.g. third leaf stage) (Song *et al.* 2019). Integrating GWAS with temporal phenotypes (TPP RGB), loci controlling the temporal VIs explained the phenotypic variations of multiple VIs revealing the pleiotropic effects of the loci. Additional candidate genes for other hotspots are discussed in the Supplementary File 1.

### Genomic prediction results of temporal phenomic data

**Fig. 2.**
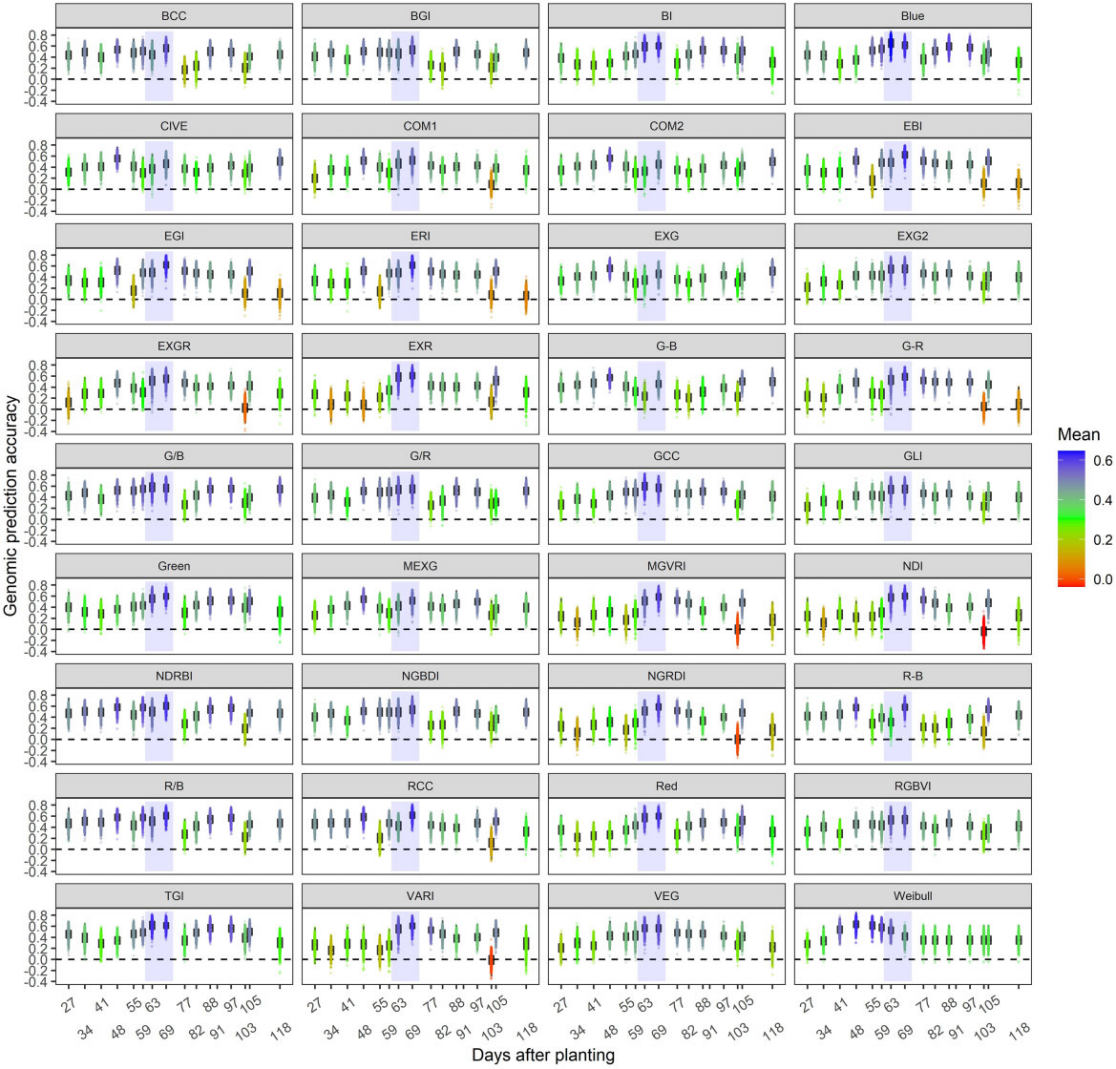
Each box plot shows the genomic prediction accuracy results belonging to each time points of each temporal trait in TPP_RGB, each contains 500-prediction accuracies. *Y* axis shows the prediction accuracy and x axis shows the flight date as days after planting time. Each box plot was colored based on the mean. Heatmap color scale was given in the figure legend changing between 0 and 0.6. Gray shading in each represents flowering time. Different time points of temporal traits were found to have different responses to genetic markers across growth stages of plant development.

GP results of temporal VI’s identified specific time points for each of the high-resolution VIs in TPP_RGB of 280 hybrids in OM had varying ability to be predicted in cross-validation (Fig. 2). Prediction accuracy showed the period of growth around flowering was the most (and in a few cases least) predictable by genomic markers for many VI’s likely because of differential emergence of tassels (Fig. 2). It was surprising that time points prior to flowering in some cases had relatively similar or higher prediction accuracy than those at flowering time (Fig. 2). Overall, sensor-based VIs were predictable at different time points using whole-genome markers but estimated different phenotypic effect sizes at each timepoint, in patterns that appear to have some biological basis (Fig. 2). Showing genetic markers estimate changing effects sizes to reveal a plasticity of temporal VIs help demonstrate why temporal VIs were more explanatory to monitor the interactions between genetic background of plants and their growing stages and growing environments throughout growth (Supplementary Fig. 9 in File 1).

### Genomic prediction vs phenomic prediction

**Fig. 3.**
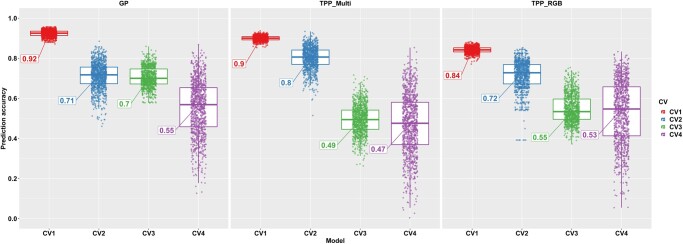
The prediction accuracy results of yield belonging to the 3 models. GP represents the prediction accuracy of genomic prediction, TPP_Multi represents the prediction accuracy of phenomic prediction using the VIs derived from the multispectral images with low resolution, TPP_RGB represents the prediction accuracy of phenomic prediction using the VIs derived from the RGB images with high resolution. Four cross-validation schemes were used: predicting tested genotypes in tested environments (CV1), predicting untested genotypes in tested environments (CV2), tested genotypes in untested environments (CV3), and untested genotypes in untested environments (CV4). Phenomic prediction predicted the grain yield (GY) of maize hybrids better in CV2 than genomic prediction. Prediction accuracies were close to each other in CV3 and CV4.

Grain yield (GY) prediction accuracy of phenomic and genomic approaches were compared between both phenomic data sets (TPP_RGB and TPP_Multi) and genomic data (GP) of the 118 hybrids with complete data. Comparing model prediction accuracies for untested genotypes in tested environment (CV2), low-resolution multispectral-related phenomic prediction (TPP_Multi) outperformed ($\bar{r}\;$= 0.80) both GP ($\bar{r}$ = 0.71) and high-resolution RGB-related phenomic prediction (TPP_RGB; $\bar{r}$ = 0.72) (Fig. 3). Comparing model prediction accuracies for untested genotypes in untested environment (CV4), GP and high-resolution RGB-related phenomic prediction supplied close prediction accuracies ($\bar{r}$: 0.53–0.55), while low resolution with multispectral sensor-based HTP supplied a lower prediction accuracy ($\bar{r}$: 0.47) (Fig. 3). Overall, the phenomic prediction platforms used in this study were largely able to predict untested genotypes in tested environment better than GP (CV2) while TPP platforms predicted untested genotypes in untested environments similarly with GP, in which TPP_Multi and TPP_RGB predicted 0.08 and 0.02 less than GP on average (Supplementary Fig. 3 in File 1). However, GP outperformed phenomic prediction when predicting known genotypes in unknown environments (CV3). Combining both UAS measures (TPP_RGB and TPP_Multi) using ridge regression did not further improve prediction accuracies with our data set (data not shown).

## Discussion

Field-based high-throughput phenotyping technologies, such as drones, are able to provide phenome-wide measurements of plants in much the same way that high-throughput sequencers have provided genome-wide data. Uniquely, phenotyping technologies can screen high numbers of plots repeatedly through the growing period resulting in not only high spatial resolution but also high temporal resolution, helping dissect how different genotypes respond to their environments to maximize fitness in near real time (Supplementary Figs. 7 and 8 in File 1). Correlations were variable between overall VIs in the phenomic data (Supplementary Fig. 9 in File 1). First, there were low to moderate correlations between VIs. Second, temporal values of the same VIs had low correlations across different time points. These results indicate that both using different VIs and their temporal values belonging to multiple time points provide unique and additional information; thus importance of including different VIs and high temporal dimension in constructing the phenomic data were emphasized.

As new temporal phenomic markers are difficult to independently measure and validate, one of the first approaches to evaluate phenomic marker utility is to look at heritability/repeatability values over different replicates and environments. This approach is not needed for genomic markers which do not vary over replicates and environments and theoretically have a repeatability near 1 but are also unable to capture environmental interaction in real time. Temporal repeatability (Supplementary Equation 3) of VIs were moderate, above ∼0.5 for TPP_RGB (Supplementary Fig. 2 in File 1) and between 0.26 and 0.66 for TPP_Multi (Supplementary Fig. 3 in File 1). Temporal repeatability relied on variation across plant development, biologically more meaningful than using genotypic variation which is static at every time point. Temporal variation captured by drones assesses temporal genotypic variation jointly over time via nested design (Supplementary Equation 2). Previously, repeatability has only been calculated between different VIs/CHM and yield at one or few time points (Rutkoski *et al.* 2016; Aguate *et al.* 2017; Montesinos-López *et al.* 2017; Anderson *et al.* 2019; Sun *et al.* 2019; Wu *et al.* 2019; Krause *et al.* 2020; Galán *et al.* 2021); disregarding the temporal genotypic variation occurring across plant growth. Furthermore, previous studies using either one or a limited number time points analyzed each time point separately.

High dimensional and temporal resolution phenomic data used in predictive plant breeding integrated with high-throughput genotyping data discovered underlying genetic causes for many important temporal VI features. For instance, pleiotropy discovered via GWAS identified specific loci controlling the AUC of many VIs (Supplementary Figs. 14 and 15 in File 1 and Dataset 2) though these VIs themselves are largely independent and uncorrelated. In addition, GP of temporal VI phenotypes proved that estimated effects of each marker varied through time, causing different prediction accuracy results for temporal phenotypes of the same VIs (Fig. 2). Therefore, instead of depending on discrete genome-wide markers as predictors for yield, temporal phenotype data formed by estimated temporal marker effects can be used to better predict certain scenarios (e.g. untested genotypes in tested environment). Predicting grain yield of untested genotypes in a tested environment is an important scenario for public breeding programs because lines developed in public breeding programs are mostly targeted for specific environments. Figure 3 showed that TPP predicted the grain yield better than GP in CV2 indicating that TPP could be a better solution for public breeding programs for genetic gain. In addition, the predictive ability of TPP in untested genotype untested environments (CV4) was in the same range as that of GS (Fig. 3). This is also an important proof of concept that TPP can be used as widely as GP. GP methods have been developed over more than a decade and phenomic prediction methods can likewise be improved. Further optimization and improvement of this approach will likely benefit from the integration of novel crop growth models as GP has Messina *et al.* (2018).

### Phenomic data can predict yield and flowering times via machine learning regressions

Penalized linear regression models using shrinkage were previously shown as the best performing prediction models when using different hyper parameters have been adapted for predicting both yield (Aguate *et al.* 2017; Kismiantini *et al.* 2021; Adak, Murray, Božinović *et al.* 2021) and flowering times (Adak, Murray, Božinović *et al.* 2021) when different reflection bands were used as predictors. Penalized linear methods with different regularization parameter settings to predict yield and flowering times (Fig. 1) were more accurate than linear regression (Montesinos-López *et al.* 2017; Adak, Murray, Božinović *et al.* 2021). This is because simple linear regression tends to overfit when there are increasing numbers of predictors and with fluctuating collinearity between predictors, such as in phenomic data. Penalized linear (ridge, lasso, and elasticnet) models or the non-linear (randomforest) model are capable to explain deviations in linear or non-linear relationships of temporal genotypic values belonging to high- or low-resolution phenomic data.

Tuning regularization parameters of the ridge, lasso and elastic net-based prediction models is a good approach to deal with model overfitting when high dimensional phenomics data are used in prediction. Tuned regularization parameters in ridge, lasso, and elastic net models can lessen coefficients, and predict test data more reliably than linear models. For example, genotype within flight combination ($\Omega_{i(j)}$ component in Supplementary Equation 2) was found to be statistically significant for all VI and CHM (Supplementary Fig. 2 in File 1) indicating a temporal interaction among the genotype across flight times because of fluctuating temporal phenotype values of VIs (Supplementary Figs. 7 and 8 in File 1). Nevertheless, a general trend demonstrated that high- and low-yielding genotypes segregate according to temporal phenotypes of VIs. A lack of correlations in temporal genotypic values of the genotype through time supports the existence of nonlinear relationships, problematic for linear models to capture. Because of multiple decision tree learning, the random forest model accounts best for non-linearity, limiting overfitting.

Phenomic prediction reached up to ∼0.80 for grain yield and flowering time prediction (Fig. 1) higher than previously reported prediction accuracies (Rutkoski *et al.* 2016; Aguate *et al.* 2017; Sun *et al.* 2019; Wu *et al.* 2019; Krause *et al.* 2020; Galán *et al.* 2021). Aguate *et al.* (2017) showed use of raw reflected bands instead of ratios (e.g. VIs) performed better in prediction models. Montesinos-López *et al.* (2017) further reported using all bands simultaneously increased prediction accuracy instead of VIs alone. However, reflected bands used in past studies derived from 5 to 9 time points, lower time dimension data than what we generated in this study. This suggests that predictors derived from additional time points could play an important role on increasing the prediction ability of the models; more so than using the predictors as either raw reflectance bands or VIs.

### Genomic prediction for temporal traits can vary depending on the time points of growth

TPP_RGB phenomic data tested using GP to identify temporal marker effects and their prediction accuracies for each VI and Weibull_CHM throughout time (Fig. 2) demonstrated that genomic markers could predict an individual’s VI or Weibull_CHM value through cross-validation using other individuals at the same stage. This demonstrated that certain stages and VIs have more genetic determination and are more heritable.

Temporally varying marker effects on the phenotype of VIs resulted in phenotypes at different timepoints of VIs and Weibull_CHM having different correlations with yield (Supplementary Figs. 5 and 6 in File 1) as well as different prediction abilities for dependent variables (Fig. 2). A dynamic pattern of marker effects as shown here has so far been overlooked in GP/selection of yield. Bernardo (2021) underlined that predicting candidate genotypes using phenotype information collected from across multiple environments may be more accurate than using the genetic markers in a prediction model. Similarly, instead of predicting grain yield fitness by whole-genome marker effect approaches such as RR-BLUP and GBLUP, including the temporal phenotypic variation occurring across growth into prediction models can result in more accurate fitness prediction as phenomic data already contain temporal marker effects. This study also showed that specific loci can explain different phenotypic variance across more than one derived VI (Supplementary Figs. 14 and 15 in File 1 and Dataset 2) signifying pleiotropic effects of certain markers for the VIs. These pleiotropic effects have various associations with developing young tissues, inflorescence, and yield.

### Phenomic prediction can perform similarly to or outperform genomic prediction

Phenomic data (TPP_Multi and TPP_RGB) predicted grain yield as well as genomic data using ridge regression (Fig. 3), but different results were observed depending on the cross-validation scheme. TPP_RGB contained 35 VIs derived from only RGB bands and Weibull_ CHM belonging to 15 time points (525 phenomic features) resulting in an accuracy of 0.71; this accuracy was same as the accuracy of 0.71 belonging to GP containing the 153,252 segregating whole-genome markers. However, when TPP_Multi, which contains the 89 VIs derived from the multispectral bands and Weibull_CHM belonging to 12 time points (1,068 phenomic features), were used in the prediction the yield, prediction accuracy reached up to 0.80; substantially higher than both GP and TPP_RGB supplied for the untested genotype in tested environments schemes (CV2) (Fig. 3). Moreover, in the most challenging cross-validation scheme, untested genotypes in untested environment (CV4), GP, TPP_RGB, and TPP_Multi performed approximately equally as their prediction accuracies were around 0.50 ± 0.05 (Fig. 3). These empirical findings suggest, for the first time, that increasing temporal as well as spectral information could be used to predict fitness substantially better than GP. This also suggests that temporal and continuous phenomic data can be better predictors than discrete genomic data in prediction and selection of high yielding genotypes. It is also important to note that hybrids used in G2F contains the high genetic diversity, it is likely to expect the high prediction accuracies that does not necessarily reflect a public or special breeding populations (Windhausen *et al.* 2012), but G2F hybrid population is still useful population to compare temporal phenomic and GPs. Successful phenomic prediction studies reported to date have used NIRS data; some of phenomic prediction results performed favorably in comparisons with GP (Rincent *et al.* 2018; Lane *et al.* 2020; Lane and Murray 2021; Zhu *et al.* 2021; Robert *et al.* 2022; Weiß *et al.* 2022; Zhu *et al.* 2022). Drone image-derived data have also been used as complementary data in GP that increased prediction accuracy of grain yield in wheat (Rutkoski *et al.* 2016; Sun *et al.* 2019; Galán *et al.* 2020; Krause *et al.* 2020). However, these studies have not used the temporal phenomic data derived from the drone images belonging to multiple time points. Multiple time point derived phenomic data have been shown in this study to predict grain yield in maize similarly or even better than GP depending on the prediction scenarios defined. Overall, phenomic selection is an emerging approach that may replace genotyping each year required by GP/selection with phenotyping. Adding a temporal component into phenomic prediction has innumerable known and yet to be discovered advantages.

In summary, this study demonstrated the predictive capability of phenomic data for complex traits in maize, yielding as much as genomic markers frequently applied in plant selection over the past 20 plus years. UAS surveys over the experimental field plots supplied temporal traits as predictors to facilitate the selection of untested genotypes in untested environments. Growing more plants and measuring them accurately are critical steps to drive effectiveness of selection intensity and accuracy resulting in higher genetic gain over time. This study exemplified that screening more plants and measuring them thanks to repetitive UAV flights across plant growth may results in greater genetic gain than genomic selection when phenomic prediction/selection is applied routinely.

## Conclusion

Genetic prediction methods, primarily genomic selection, became instrumental over the last decade to drive genetic gain for crop improvement. Such prediction methods leverage information shared between relatives to predict an individual’s fitness but remain prohibitively resource intensive and unable to dissect responses to a changing environment. UAS (i.e. drones with sensors) have demonstrated high-throughput, low-resource approaches to temporally evaluate fitness of large and genetically diverse populations. For the first time, this study demonstrates that TPP made from UAS have capacity to perform equal to or better than genomic selection and require fewer resources. TPP success opens new lines of inquiry for understanding organism reactions to their environment and for our understanding of genetic relationships.

## Supplementary Material

jkac294_Supplementary_Data

## Data Availability

Supplementary Dataset 1 contains the 4 phenomic data that belongs to RGB HTP platform in optimal management (TPP_RGB_OM), RGB HTP platform in stress management (TPP_RGB_SM), multispectral HTP platform in optimal management (TPP_Multi_OM), and multispectral HTP platform in stress management (TPP_Multi_SM). Supplementary Dataset 2 contains the discovered SNPs in GWAS for the AUC phenotype values of each VI along with their chromosome, chromosome positions, *P*-values, minor allele frequencies, effects, explained % variation, Vis, and GWAS models. Supplemental material is available at G3 online.
