# Supplementary material for: Temporal phenomic predictions from unoccupied aerial systems can outperform genomic predictions
Source: G3 (Bethesda). 2022 Nov 29;13(1):jkac294. doi: 10.1093/g3journal/jkac294 (PMC9836347; doi:10.1093/g3journal/jkac294)
Supplement: jkac294_Supplementary_Data [file jkac294_supplementary_data.zip › Suppl/File_S1_G3-2022-403168.docx]

**Supporting Information (SI appendix)**

Adak *et al.* Temporal unoccupied aerial system phenomic predictions can outperform genomic predictions

**SI Materials and Methods**

**Phenomic data extraction pipeline**

Detailed function settings of R/UAStools::plotshpcreate were set as follows: (i) *nrowplot* was set 2 since two consecutive row plots represent the one hybrid genotype, *multirowind* was also set TRUE (T) to define two consecutive row plots indicates one genotype; (ii) dimension of each polygon was defined by setting the functions of *rangespc* and *rowspc* as 7.62 and 0.76 meters respectively; (iii) buffer polygon was obtained by removing the alley distances from left, right, top and bottom sides using *rangebuf* and *rowbuf* functions; buffer polygon was obtained by setting *rangebuf* (for top and bottom sides) and *rowbuf* (for left and right sides) as 0.61 and 0.05 meters respectively. Buffer polygons covering each plot were used as shape files in data extraction pipelines to obtain better accuracy since walking alleys surrounding the plots were excluded (<https://github.com/andersst91/UAStools/wiki/plotshpcreate.R>). As a result, a shape file containing 594 buffer polygons (each contain two row plots) were created with the unique plot number in each. After constructing the shape file, each buffer polygon was visualized with tiff files for each time point in QGIS software (<https://qgis.org/en/site>) and checked manually; occasionally a small percentage of polygons was required to move slightly around the row plots to make each cover the row plots accurately because of the minor overlap issue for certain region of the mosaicked tiff files~~.~~

To extract the VIs, first, the tiff files were clipped into a trial level in QGIS, then the extraction pipeline was applied to each clipped tiff file in R. Extraction pipeline were explained briefly as follows: (i) *aggregate* function was first implemented to each tiff file consistently to reduce the computational time requirement by setting *fact* as 4 [*aggregate(“input tiff file”, fact = 4*]; (ii) soil was removed from the tiff files by using the Hue index in *R/FIELDImageR::fieldMask* function; (Escadafal 1993); (iii) additional VIs for both HTP platforms were defined in *R/FIELDImageR::fieldndex* function using the output tiff file of second step; (iv) previously constructed shape files were combined with the output of the third step to obtain the values of each VI for each row plot. The VIs calculated by using RGB bands were extracted from the images in low altitude high resolution RGB HTP platform while VIs calculated by using RGB, red edge and NIR bands were extracted from the images in high altitude lower resolution multispectral HTP platform.

To construct the canopy height model (CHM), each 3D point cloud file (.las) was first clipped into trial level then the following steps of the custom batch code was applied to each point cloud to extract the plot based temporal plant height as follows: (i) sorting the clipped point clouds to facilitate further processing steps (LAStools/lasssort.exe); (ii) removing excessively noise points (blunders) located below ground and above canopy (LAStools/lasnoise.exe) of row plots; (iii) using the hierarchical robust interpolation algorithm (HRI) (Kraus and Pfeifer 1998) to determine the ground points (FUSION\GroundFilter.exe); (iv) detecting the key points from the ground filter to outline digital terrain model (DTM) (LAStools\lasthin.exe); (v) creating the DTM model using the key points from the previous step (FUSION\GridSurfaceCreate.exe); (vi) generating the canopy surface model by extracting the digital terrain model (output of step v) from the digital surface model (output of step ii) (LAStools\lasheight.exe). Adjusting ‘Z’ values that account for plant height in the canopy surface model, merging with the ESRI shape file to clip the canopy surface model into plots (FUSION/PolyClipData.exe). As a last step, statistical metrics (e.g., plant height values based on different percentiles) for each clipped plot (genotype row) were calculated (FUSION/CloudMetrics.exe). Predicted CHM for each genotype by equation 2 (*Eq. 2*) was fit based on the Weibull sigmoidal growth model as follows:

$$Weibull sigmoidal growth= a\left( 1-Exp\left( -\left( \frac{flight date}{x} \right)^{b} \right) \right) Equation 1;Eq. 1$$

Where, $a$ is the asymptote; $flight date$ is numeric values as days after planting of each flight date; $x$ is the inflection point and $b$ is the growth rate. Weibull fit CHM was used in further analysis.

**Experimental design and nested model for phenomic data**

Following extraction of plot based temporal vegetation indices and CHM, a nested design predicted the temporal genotypic values for each of 280 genotype to assess the temporal phenomic data jointly for optimal management (OM) and stressed management (SM, no irrigation, low fertilizer) using the “lmer” function in the “lme4” package in R. Each temporal vegetation index and temporal plant height was modelled for both HTP platform as follows:

$$Y_{ijklm}=\mu+\beta_{i}+\Omega_{i(j)}+\delta_{i(k)}+\Psi_{i(l)}+\theta_{i(m)}+ɛ_{ijklm} (Equation 2;Eq.2)$$

where, $\mu$ = overall mean; $\beta_{i}$ = the random effect of $i$th flight time (as days after planting time, DAP) with $\beta_{i} {iid \atop\sim} N(0, \sigma_{\beta_{i}}^{2})$, $i \in\{$27, 34, 41, 48, 55, 59, 63, 69, 77, 82, 88, 97, 103, 105, 118; rotary-wing with RGB camera HTP platform} and $i \in\{$27, 34, 52, 60, 70, 73, 88, 105, 112, 118, 132, 144; tuffwing with multispectral camera HTP platform}; $\Omega_{i(j)}$= the random effect of $j$th genotype (maize hybrid) within the $i$th flight time with $\Omega_{i(j)} {iid \atop\sim} N(0, \sigma_{\Omega_{i(j)}}^{2})$, $j\in\{1, \ldots, 280\}$; $\delta_{i(k)}$ = the random effect of $k$th range within the $i$th flight time with $\delta_{i(k)} {iid \atop\sim} N(0, \sigma_{\delta_{i(k)}}^{2})$, $k\in\{1, \ldots, 18\}$; $\Psi_{i(l)}$= the random effect of $l$th row within the $i$th flight time with $\Psi_{i(l)} {iid \atop\sim} N(0, \sigma_{\Psi_{i(l)}}^{2})$, $l\in\{1, \ldots, 33\}$; $\theta_{i(m)}$= the random effect of $m$th replication within the $i$th flight time with $\theta_{i(m)} {iid \atop\sim} N(0, \sigma_{\theta_{i(m)}}^{2})$, $m\in\{1, 2\}$; $ɛ_{ijklm}$ is pooled error with $ɛ_{ijklm}{iid \atop\sim} N\left( 0, \sigma_{ɛ_{ijklm}}^{2} \right).$

Temporal repeatability (TR) was calculated using the genotypic variance containing the variation of the trait belonging to all flight times. *Eq. 3* was applied to each vegetation index and canopy height separately.

$$Temporal repeatability (TR)=\frac{\sigma_{\Omega_{i\left( j \right)}}^{2}}{\sigma_{\Omega_{i\left( j \right)}}^{2}+\frac{\sigma_{ɛ_{ijklm}}^{2}}{no. of replications}} (Equation 3;Eq. 3)$$

Where, $\sigma_{\Omega_{i\left( j \right)}}^{2}$ is genotypic variance containing joint genotypic variation occurring across the flights; $\sigma_{ɛ_{ijklm}}^{2}$ is residual variance containing unexplained error.

Grain yield (GY) was collected from each two adjacent row plots (per hybrid) via a plot combine harvester; days to anthesis (DTA) and silking (DTS) were collected when fifty percent of plots displayed anthesis and silking emergence; manually measured terminal plant height was calculated from the ground to the tip of tassel.

GY, DTA, DTS, PHT were used as predicted variables and modelled according to *Eq. 2* without flight time (denoted as$\beta$ in *Eq. 2*) component as follows:

$$Y_{jklm}=\mu+\Omega_{j}+\delta_{k}+\Psi_{l}+\theta_{m}+ɛ_{jklm} (Equation 4;Eq.4)$$

Traditional repeatability was calculated for all cumulative traits (GY, DTA, DTS, PHT) based on *Eq. 3* with the nested effect by flight time removed (denoted as$\beta$ in *Eq. 2*) as follows:

$$Repeatability=\frac{\sigma_{\Omega_{j}}^{2}}{\sigma_{\Omega_{j}}^{2}+\frac{\sigma_{ɛ_{jklm}}^{2}}{no. of replications}} (Equation 5;Eq.5)$$

As a result of $\Omega_{i(j)}$ component in $Eq. 2$, 35 VIs and Weibull_CHM belonging to fifteen time points in TPP_RGB, and 89 VIs and Weibull_CHM belonging to twelve time points in TPP_Multi were predicted, resulting in 540 and 1080 phenomic data features belonging to the 280 maize hybrids. Pearson correlation coefficients between each phenotype data feature at each time point of each temporal trait with GY were calculated using the “*corrplot*” package in R.

**Machine learning based phenomic prediction models**

*Caret* package was used in R to run the prediction models (Bates et al. 2014). “*Caret::trainControl()*” function was used to set repeated cross validation (*method=“repeatedcv”*) with 10 folds and 3 repeats; this cross validation was used for every model consistently inside the loop. Brief steps of the single loop were explained as follows: (i) partitioning the whole data set as 70 percent training data with the remaining 30 percent as a test data set in TPP_RGB and TPP_Multi phenomic data belonging to optimal (OM) and stress (SM) managements, which were different in each loop, using the “*caret::createDataPartition()*” function, (ii) training the all prediction models using the train data set of OM (tested environment) in the “*caret::train()*” function, (iii) predicting the train data set in OM (cross validation 1; tested genotypes in tested environment; CV1), test data set in OM (cross validation 2; untested genotypes in tested environment; CV2), train data set in SM (cross validation 3; tested genotypes in untested environment; CV3) and test data set in OM (cross validation 4; untested genotypes in untested environment; CV4) using the trained model to obtain the predicted data using the “*caret::predict()*” function for each model, (iv) computing the correlation between actual data and predicted data to evaluate the prediction accuracy $(\bar{r})$for four cross validation schemes in each model and (v) saving the correlation results along with the R-squared (R^2^), root mean square error (RMSE) and mean absolute error (MAE) as well as the variable importance scores of the predictors belonging to each model in each loop. Number of loops was set to 500. To define the prediction model inside the “*caret::train()*” function, method was set as “*lm*” for the linear model, method was set as “*glmnet*” for elastic net, lasso and ridge models and model was set as “*rf*” for random forest (RF) model separately. To tune the parameters of the elastic net, lasso and ridge regressions, “*alpha*” value was set as 0 for ridge and 1 for lasso regression while sequential numbers between 0 and 1 by ten equal increment numbers were searched to find the best alpha for elastic net regression. Sequential “*lambda*” values between 0 and 1 by ten equal increment numbers were also searched to find the best “*lambda*” values for lasso, ridge, and elastic net regressions. To tune the parameters of RF model, “*ntree*” (number of trees to grow in the model) was set as 1000 while sequential “*mtry*” (number of variables randomly tested as candidates at each split) value between 1 and 50 by five equal increment number were searched to find the best “*mtry*” based on highest accuracy metric of RF model. *varImp* function was used to extract the variable importance scores in caret package for each regression model.

**Association mapping for phenomic data**

Cumulative AUC were calculated by using the below formula for each genotype and each VI and Weibull_CHM:

$$Cumulative {AUC}_{i}=\sum_{t=1}^{n-1} \left( \frac{V_{t}+V_{t+1}}{2} \right)(F_{t+1}-F_{t}) (Equation 6; Eq. 6)$$

Where, $n$is the number of total observations referring to the fifteen flights times in TPP_RGB, $Cumulative {AUC}_{i}$ is the cumulative AUC value based on the total number of flights belonging to $ith$ genotype for each trait, $i\in\{1,\ldots, 280\}$; $V_{t}$ is the value at $t$th flight time as DAP,$t\in\{27, 34, 41, 48, 55, 59, 63, 69, 77, 82, 88, 97, 103, 105, 118\}$; $F_{t}$ is the $t$th flight time as number of DAP at which value of interest was taken in first HTP platform.

The imputed ZeaGBSv2.7 with AGPv4 coordinates was used in this study, available in Panzea (<https://www.panzea.org/genotypes>) and Cyverse (McFarland et al. 2020) platforms. In the genome wide association mapping study, 158 maize hybrids with genotyping by sequencing (GBS) data of their parental lines were available in MaizeGBSv2.7 (Glaubitz et al. 2014), generated via the method based on digestion the DNA with the *ApeKI* restriction enzyme (Elshire et al. 2011). ZeaGBSv2.7 was called in Tassel 5 software (Bradbury et al. 2007). Before association mapping, GBS data of hybrid maize was created based on following step: (i) heterozygote calls belonging to any parental lines of the hybrids were set as missing, (ii) “*create_hybrid_genotype*” function in Tassel 5 software was used to create the GBS data of hybrid maize by merging the GBS data of parental lines of each hybrid and (iii) polymorphic markers were obtained by filtering the missing data that is more than ten percent and minor allele frequency that is lower than five percent. LD *k*-nearest neighbor algorithm (LD *KNNi* imputation) was implemented to GBS data to impute the missing calls in Tassel software (version 5) (Money et al. 2015). Finally, 101,100 polymorphic SNPs (single nucleotide polymorphism) remained and were used in the association mapping analysis.

To control the population structure of the hybrid population, the first five principal components, which explained 49% of total variation, and kinship matrix were used in each model. Bonferroni corrections [$-{log}_{10} \left( p values \right) >6.3$; 0.01/(no. of markers)] were considered as threshold in determining the GWAS hits in Manhattan plots, in addition to Bonferroni threshold, false-positive discovery rate was set [$-{log}_{10} \left( p values \right)>5$] to detect same loci (if any) that were associated with multiple traits with between the values of [$-{log}_{10} \left( p values \right) >5]$ and [$-{log}_{10} \left( p values \right)>6.3]$. MaizeGBD (<http://www.maizegdb.org/>) and the Gramene database ([http://www.gramene.org](http://www.gramene.org/)) were used to determine corresponding candidate genes of the discovered SNPs and functions of genes. LD decay pattern was investigated in Tassel 5 (LD windows size = 10 markers) and visualized in R for each chromosome separately (**Fig. S1**). Linkage disequilibrium (LD) was visualized using the *LDheatmap* package in R (Shin et al. 2006) to identify the candidate genes within the LD blocks ($R^{2}$ ≥ 0.8) of colocalized SNPs.

**Genomic prediction for phenomic data**

153,252 SNPs belonging to 158 maize hybrids were obtained merging the GBS data of their parental lines in Tassel software as described in the “*Association mapping for phenomic data”* section. After obtaining the hybrid GBS, SNPs were filtered if minor allele frequency was lower than 0.01 and missing values were higher than 10 percent per marker resulting in 153,252 SNPs. Missing values of 153,252 SNPs were imputed using the *rrBLUP::Amat()* function in R. Temporal genomic prediction for phenomic data in TPP_RGB was modeled using the rrBLUP package (Endelman 2011) in R as follows:

$$y=1\mu+ZФ+ɛ (Equation 7;Eq. 7)$$

Where, $y$ = is the vector $(n \times1)$ of single phenotype data of $n$ maize hybrids ($n$ = is training data set of each loop) belonging to each single time point of each phenotype data in TPP_RGB , $\left[ \begin{aligned} 0.6 \\ 0.9 \\ \vdots\\ n \end{aligned} \right]$; $1=$ vector of ones that are equal to numbers of $n$, $\left[ \begin{aligned} 1 \\ 1 \\ \vdots\\ n \end{aligned} \right]$; $\mu$ = overall mean of training data set; $Z$ = the incidence matrix $(n \times p)$ of allelic states of $p$ number of SNPs (153,252 SNPs) belonging to $n$ number of maize hybrids, $\left[ \begin{matrix} 0 & 1\cdots& 0 \\ \vdots& \ddots& \vdots\\ -1 & 0\cdots& n\times p \end{matrix} \right]$; $Ф$ = vector of calculated SNP effects $(p \times1)$, $\left[ \begin{aligned} 4e-05 \\ -5e-05 \\ \vdots\\ p \end{aligned} \right];$ $ɛ$ = vector of random residuals. RR-BLUP assumes $Ф \sim N(0,\sigma_{Ф}^{2})$ indicating that marker effects are normally distributed with equal marker variance $(\sigma_{Ф}^{2})$ throughout the genome.

Genomic prediction was modelled using training data accounting for seventy percent of total data while the remaining thirty percent data was used as test data. The genomic prediction model ($Equation 7, Eq. 7$) evaluated 500 iterations applied to each phenotype of 158 maize hybrids belonging to each VI and Weibull_CHM at fifteen time points (in total 540 phenotypic features) in TPP_RGB; base R function called “*sample()*”was used to randomly determine the training and test data set in each iteration. During the prediction, the same training and test data set for each phenotype of each trait at each time point was needed to obtain fair comparison of the genomic prediction accuracy. Genomic prediction accuracy was calculated based on correlation results between the genetic estimated breeding values and the true breeding value of the test data set in each iteration.

**Phenomic prediction versus genomic prediction**

118 maize hybrids whose parental lines had GBS info in MaizeGBSv2.7 (Glaubitz et al. 2014) and grown in optimal (OM) and stressed (SM) managements were used. In genomic prediction, GBS data (GP) containing 153,252 SNPs that was described in the “*Genomic prediction for phenomic data”* was used to predict grain yield (GY) using rrBLUP package in R (Endelman 2011). TPP_RGB containing 540 phenomic data, and TPP_Multi containing 1080 phenomic data were used to predict GY using the ridge regression in the *caret/glmnet* package in R. Prediction accuracy was obtained from 1000 bootstraps for each model where the same training and test data set were used for genomic and phenomic prediction within each bootstrap. Genomic prediction and phenomic prediction steps were explained in the *“Genomic prediction for phenomic data”* and *“Machine learning based phenomic prediction models”* respectively. Four cross validation schemes, which were explained in *“Machine learning based phenomic prediction models”***,** were applied in this section as well to compare the prediction accuracies of two phenomic predictions and genomic prediction.

**Table S1** shows the calendar days between March to July, 2017 containing the flight dates for the low altitude high resolution rotary-wing UAS with RGB camera (above) and the high altitude lower resolution TuffWing UAS with multispectral camera (below) high throughput phenotyping platforms. Flight dates were shown under the months with corresponding days after planting times in parenthesis. All VIs were calculated at each time point for each hybrid in that VIs used in RGB and Multispectral high throughput phenotyping platforms were given in Table S2

| **Rotary-wing with RGB camera** | | | | | | | | | | | | | | | | | | | | | |
| --- | --- | --- | --- | --- | --- | --- | --- | --- | --- | --- | --- | --- | --- | --- | --- | --- | --- | --- | --- | --- | --- |
| March | April | | | | | | | May | | | | | | | | | June | | | | |
| 29^th^  (27) | 5^th^  (34) | | 12^th^  (41) | 19^th^  (48) | | 26^th^  (55) | 30^th^  (59) | 4^th^  (63) | | 10^th^  (69) | | 18^th^  (77) | | 23^rd^  (82) | | 29^th^  (88) | 7^th^  (97) | 13^th^  (103) | 15^th^  (105) | 28^th^  (118) | |
| **TuffWing with multispectral camera** | | | | | | | | | | | | | | | | | | | | | |
| March | | April | | | | | | | May | | | | | | | | June | | | July | |
| 29^th^  (27) | | 5^th^  (34) | | | 23^rd^  (52) | | | | 1^st^  (60) | | 11^th^  (70) | | 14^th^  (73) | | 29^th^  (88) | | 15^th^  (105) | 22^nd^  (112) | 28^th^  (118) | 12^th^  (132) | 24^th^  (144) |

**Table S2** shows the vegetation indices used and their formulas along with references.

| Vegetation index | Ratios | References |
| --- | --- | --- |
| **VIs derived from RGB bands** | | |
| Blue chromatic coordinate index (BCC) | $\frac{B}{R+G+B}$ | (Woebbecke et al. 1995) |
| Blue green pigment index (BGI) | $\frac{B}{G}$ | (Zarco-Tejada et al. 2005) |
| Brightness index (BI) | $sqrt(\frac{R^{2}+G^{2}+B^{2}}{3})$ | (Richardson and Wiegand 1977) |
| Color index of vegetation extraction (CIVE) | $0.441R-0.811G+$  $0.385B+18.78745$ | (Kataoka et al. 2003) |
| Combined indices 1 (COM1) | $EXG+CIVE+EXGR+VEG$ | (Guijarro et al. 2011) |
| Combined indices 2 (COM2) | $0.36EXG+0.47CIVE+0.17VEG$ | (Guerrero et al. 2012) |
| Additional blue index (EBI) | $\frac{B-G}{B-R}$ | (Golzarian and Frick 2011) |
| Additional green index (EGI) | $\frac{G-R}{R-B}$ | (Golzarian and Frick 2011) |
| Green-red index (ERI) | $\frac{R-G}{R-B}$ | (Golzarian and Frick 2011) |
| Excessive green (EXG) | $2G-R-B$ | (Woebbecke et al. 1995) |
| Normalized Excess green index (EXG2) | $\frac{2G-R-B}{G+R+B}$ | (Woebbecke et al. 1995) |
| Excess green minus excess red index (EXGR) | $3G-2.4R-B$ | (Meyer and Neto 2008) |
| Excessive red (EXR) | $1.4R-G$ | (Meyer et al. 1998) |
| Green minus blue index (G-B) | $G-B$ | (Woebbecke et al. 1995) |
| Green minus red index (G-R) | $G-R$ | (Woebbecke et al. 1995) |
| Green blue simple ratio index (G/B) | $\frac{G}{B}$ | (Woebbecke et al. 1995) |
| Green red simple ratio index (G/R) | $\frac{G}{R}$ | (Woebbecke et al. 1995) |
| Green chromatic coordinate index (GCC) | $\frac{G}{R+G+B}$ | (Woebbecke et al. 1995) |
| Green leaf index (GLI) | $\frac{2G-R-B}{2G+R+B}$ | (Louhaichi et al. 2001) |
| Modified excess green index (MEXG) | $1.262G-0.884R$  $-0.311B$ | (Burgos-Artizzu et al. 2011) |
| Modified green red index (MGVRI) | $\frac{G^{2}-R^{2}}{G^{2}+R^{2}}$ | (Bendig et al. 2015) |
| Normalized difference index (NDI) | $128*[\left( \frac{\left( G-R \right)}{\left( G+R \right)} \right)+1]$ | (Meyer and Neto 2008) |
| Normalized difference red  blue index (NDRBI) | $\frac{R-B}{R+B}$ | (Golzarian and Frick 2011) |
| Normalized green-blue difference index (NGBDI) | $\frac{G-B}{G+B}$ | (Hunt et al. 2005) |
| Normalized green red difference index (NGRDI) | $\frac{G-R}{G+R}$ | (Tucker 1979) |
| Red minus blue index (R-B) | $R-B$ | (Woebbecke et al. 1995) |
| Red blue simple ratio index (R/B) | $\frac{R}{B}$ | (Woebbecke et al. 1995) |
| Red chromatic coordinate index (RCC) | $\frac{R}{R+G+B}$ | (Woebbecke et al. 1995) |
| Red green blue index (RGBVI) | $\frac{G^{2}-R*B}{G^{2}+R*B}$ | (Bendig et al. 2015) |
| Triangular greenery index (TGI) | $G-(0.39R-0.69B)$ | (Hunt et al. 2011) |
| Visible atmospherically resistant index (VARI) | $\frac{G-R}{G+R-B}$ | (Gitelson et al. 2002) |
| Vegetativen (VEG) | $\frac{G}{R^{0.667}*B^{0.334}}$ | (Hague et al. 2006) |
| **VIs derived from multispectral bands (RGB, red-edge and NIR bands)** | | |
| Modified chlorophyll absorption in reflectance index 1(MCARI1) | $\left[ \left( \mathrm{NIR}- \mathrm{RE} \right)- 0.2 *\left( \mathrm{NIR}- G \right) \right]*\left( \frac{\mathrm{NIR}}{\mathrm{RE}} \right)$ | (Daughtry et al. 2000) |
| Modified chlorophyll absorption in reflectance index 2(MCARI2) | $\frac{1.5\left( NIR-RE \right)-1.3\left( NIR-G \right)}{\sqrt{\left( 2NIR+1 \right)^{2}-\left( 6NIR-5\sqrt{\mathrm{RE}} \right)-0.5}}$ | (Haboudane et al. 2004) |
| Chlorophyll vegetation index-green (CIG) | $\frac{\mathrm{NIR}}{G}-1$ | (Gitelson et al. 2005) |
| Chlorophyll vegetation index-red edge (CIRE) | $\frac{\mathrm{NIR}}{\mathrm{RE}}-1$ | (Gitelson et al. 2005) |
| Chlorophyll vegetation index (CVI) | $\frac{NIR*R}{G^{2}}$ | (Vincini et al. 2008) |
| Difference vegetation index (DVI) | $NIR-RE$ | (Tucker 1979) |
| Enhanced normalized difference vegetation index (ENDVI) | $\frac{NIR+G-2B}{NIR+G+2B}$ | Maxmax 2015  (<http://www.maxmax.com/endvi.htm>) |
| Enhanced vegetation index (EVI) | $\frac{2.5\left( NIR-R \right)}{NIR+6R-7.5B+1}$ | (Huete et al. 2002) |
| Green difference vegetation index (GDVI) | $NIR-G$ | (Tucker 1979) |
| Green infrared percentage vegetation index (GIPVI) | $\frac{\mathrm{NIR}}{NIR+G}$ | (Crippen 1990) |
| Green normalized difference vegetation index (GNDVI) | $\frac{NIR-G}{NIR+G}$ | (Gitelson et al. 1996) |
| Green optimal soil adjusted vegetation index (GOSAVI) | $\frac{\left( 1+0.16 \right)\left( NIR-G \right)}{NIR+G+0.16}$ | (Rondeaux et al. 1996) |
| Green re-normalized different vegetation index (GRDVI) | $\frac{NIR-G}{\mathrm{sqrt}\left( NIR+G \right)}$ | (Roujean and Breon 1995) |
| Green ratio vegetation index (GRVI) | $\frac{\mathrm{NIR}}{G}$ | (Buschmann and Nagel 1993) |
| Green soil adjusted vegetation index (GSAVI) | $1.5\left( \frac{NIR-G}{NIR+G+0.5} \right)$ | (Sripada et al. 2006) |
| Green wide dynamic range vegetation index (GWDRVI) | $\frac{0.12NIR-G}{0.12NIR+G}$ | (Gitelson 2004) |
| Modified double difference index (MDD) | $(NIR-RE)-(RE-G)$ | (Le Maire et al. 2004) |
| Modified GSAVI (MGSAVI) | $0.5[2NIR + 1 - sqrt((2NIR + 1)^{2}- 8(NIR - G))]$ | (Qi et al. 1994) |
| Modified normalized difference index (MNDI) | $(NIR-RE)/(NIR-G)$ | (Datt 1999) |
| Modified normalized difference red edge (MNDRE) | $\frac{\left[ NIR-\left( RE-2G \right) \right]}{\left[ NIR+\left( RE-2G \right) \right]}$ | (Wang et al. 2012) |
| Modified RESAVI (MRESAVI) | $0.5 [2NIR + 1 - sqrt((2NIR + 1)^{2}- 8(NIR - RE))]$ | (Qi et al. 1994) |
| Modified RETVI (MRETVI) | 1.2[1.2(NIR − G) − 2.5(RE − G)] | (Haboudane et al. 2004) |
| Modified simple ratio (MSR) | $\frac{\left( \frac{\mathrm{NIR}}{R}-1 \right)}{\sqrt{\left( \frac{\mathrm{NIR}}{R}+1 \right)}}$ | (Chen 1996) |
| Modified green simple  ratio (MSR_G) | $\frac{\left( \frac{\mathrm{NIR}}{G}-1 \right)}{\sqrt{\left( \frac{\mathrm{NIR}}{G}+1 \right)}}$ | (Chen 1996) |
| Modified green simple  ratio (MSR_RE) | $\frac{\left( \frac{\mathrm{NIR}}{\mathrm{RE}}-1 \right)}{\sqrt{\left( \frac{\mathrm{NIR}}{\mathrm{RE}}+1 \right)}}$ | (Chen 1996) |
| Modified transformed  CARI (MTCARI) | $3\left[ \left( \mathrm{NIR}- \mathrm{RE} \right)- 0.2\left( \mathrm{NIR}- G \right)\left( \frac{\mathrm{NIR}}{\mathrm{RE}} \right) \right]$ | (Haboudane et al. 2002) |
| Normalized difference  red edge (NDRE) | $\frac{\mathrm{NIR}- \mathrm{RE}}{NIR+ \mathrm{RE}}$ | (Barnes et al. 2000) |
| Normalized difference vegetation index (NDVI) | $\frac{\mathrm{NIR}- R}{NIR+ R}$ | (Tucker 1979) |
| Normalized NIR index (NNIR) | $\frac{\mathrm{NIR}}{NIR+RE+ G}$ | (Sripada et al. 2006) |
| Normalized red edge index (NREI) | $\frac{\mathrm{RE}}{NIR+RE+ G}$ | (Sripada et al. 2006) |
| Normalized green index (NGI) | $\frac{G}{NIR+RE+ G}$ | (Sripada et al. 2006) |
| Optimized soil-adjusted vegetation index (OSAVI) | $\frac{NIR-R}{NIR+R+0.16}$ | (Rondeaux et al. 1996) |
| Plant senescence reflectance index (PSRI) | $\frac{R-G}{\mathrm{RE}}$ | (Merzlyak et al. 1999) |
| Red edge green difference vegetation index (REGDVI) | $RE-G$ | (Tucker 1979) |
| Red edge GNDVI (REGNDVI) | $\frac{RE-G}{RE+G}$ | (Gitelson et al. 1996) |
| Red edge green ratio vegetation index (REGRVI) | $\frac{\mathrm{RE}}{G}$ | (Cao et al. 2013) |
| Red edge optimal soil adjusted vegetation index (REOSAVI) | $\frac{\left( 1+0.16 \right)\left( NIR-RE \right)}{NIR+RE+0.16}$ | (Rondeaux et al. 1996) |
| Renormalized difference vegetation index (RERDVI) | $\frac{NIR-RE}{\sqrt{NIR+RE}}$ | (Roujean and Breon 1995) |
| Red edge soil adjusted vegetation index (RESAVI) | $1.5\left[ \frac{NIR- RE}{NIR + RE + 0.5} \right]$ | (Sripada et al. 2006) |
| Red edge transformed vegetation index (RETVI) | 0.5[120(NIR − G) − 200(RE − G)] | (Broge and Leblanc 2001) |
| Red edge wide dynamic range vegetation index (REWDRVI) | $\frac{0.12NIR-RE}{0.12NIR+RE}$ | (Gitelson 2004) |
| Ratio vegetation index (RVI) | $\frac{\mathrm{NIR}}{R}$ | (Jordan 1969) |
| Soil-adjusted vegetation index (SAVI) | $\frac{1.5\left( NIR-R \right)}{NIR+R+0.5}$ | (Huete 1988) |
| Triangular vegetation index (TVI) | 0.5[120(NIR − G) − 200(R − G)] | (Broge and Leblanc 2001) |
| Optimized vegetation  index 1 (VIopt1) | 100(lnNIR – lnRE) | (Jasper et al. 2009) |
| Transformed Normalized Vegetation Index (TNDVI) | $\mathrm{sqrt}\left( \frac{NIR-R}{NIR+R}+0.5 \right)$ | (Sandham and Zietsman 1997) |
| Modified Nonlinear Index (MNLI) | $\frac{1.5\left( NIR^{2}-R \right)}{NIR^{2}+R+0.5}$ | (Gong et al. 2003) |
| Red Edge Simple Ratio (RESR) | $\frac{RE}{R}$ | (Erdle et al. 2011) |
| Red edge normalized difference vegetation index (RENDVI) | $\frac{RE-R}{RE+R}$ | (Elsayed et al. 2015) |
| Normalized NIR index2 (NNIR2) | $\frac{\mathrm{NIR}}{NIR+RE+ R}$ | (Sripada et al. 2006) |
| Normalized red edge index2 (NREI2) | $\frac{\mathrm{RE}}{NIR+RE+ R}$ | (Sripada et al. 2006) |
| Normalized red index (NRI) | $\frac{R}{NIR+RE+ R}$ | (Sripada et al. 2006) |

R, G, B, RE and NIR represent the red, green, blue, red-edge and near infrared reflectance bands respectively. Red, green, blue, red edge and NIR reflectance bands were also used in this study singly.

**SI Results**

**Genome wide association mapping results**

Two close large effect loci 39,906,105 bp (*chr2_2*) and 39,906,547 bp (*chr2_3*) genomic locations were discovered for EXG2, GLI, RGBVI, VEG, Blue, GCC and NDRBI by all three GWAS models consistently and explained between 6 and 51 percent of the phenotypic variation depending on the traits and GWAS models (**Dataset S1**). *GRMZM2G129493* (chr2: 39906034 to 39907044 bp) candidate gene covers *chr2_2* (39906105 bp) and *chr2_3* (39906547 bp) GWAS peaks in its genomic region; known as polygalacturonase-inhibiting proteins (*PGIPs*) these encode plant defense related proteins (Ferrari et al. 2003). Another candidate gene (~2kb away from *chr2_2 and chr2_3*), *GRMZM2G362362* (chr2: 39893309 to 39904028), belongs to a family of glycoside hydrolases that hydrolase the glycosidic bonds in polysaccharide in cell wall (Minic 2008).

The 50,705,765 bp (*chr2_4*) genomic location in chromosome 2 was discovered for TGI, Blue, BI, Green and Red by all three models consistently and explained 6 to 7 percent variation depending on the traits and models (**Dataset S1**). GRMZM2G018059 (chr2: 50696420 to 50706825) candidate gene contains the *chr2_4* GWAS peak in its genomic region and its function is related to U-box domain-containing protein kinase family protein that was discovered in previous association mapping studies as drought responsive genes (Wang et al. 2016; He et al. 2020) as well as for yield related traits in maize (Zhou et al. 2020).

203,544,095 bp (*chr4_1*) genomic location in chromosome 4 was discovered for TGI, BCC, BGI, G/B, G/R, GCC, NDRBI, NGBDI, R/B, COM1, EXG2, GLI and RGBVI and explained between 3 to 44 percent variation depending on the traits and models (**Dataset S1**). The *GRMZM2G001541* (chr4: 203544095 to 203547230 bp) candidate gene is closest and ~30 base pairs away from the *chr4_1* (203544095 bp). The homolog of this gene in *Arabidopsis* is responsible for encoding the inflorescence and root apices receptor-like kinase (IRK) protein that is crucial for maintaining the differentiation of meristem (Hattan et al. 2004). *GRMZM2G001541* has been discovered by meta-QTL and GWAS analysis consistently and found to be highly expressed in developing tissues (e.g. primordia, developing leaves and ear) closely related to inflorescence development (Wu et al. 2016) influencing yield performance directly in maize (Wang et al. 2020). *GRMZM2G001541* governs the expression level of *Unbranched3* (UB3), which regulates the quantitative variation of kernel row number in maize (Liu et al. 2015).


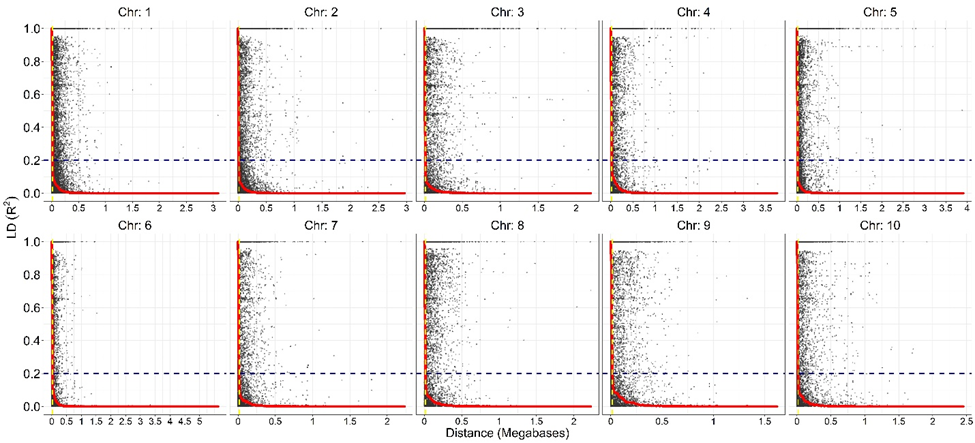


**Figure S1** shows linkage disequilibrium decay (LD) patterns for each chromosome. The Y axis represents the R^2^ while X axis shows the distance as Megabases. Horizontal dashed line shows the 0.2 R^2^ LD while the vertical yellow color dashed line shows the 15 kilo base pair (kb) threshold in each chromosome. LD decay was found to be rapid for all chromosome and changing between 1 to 15 kb.


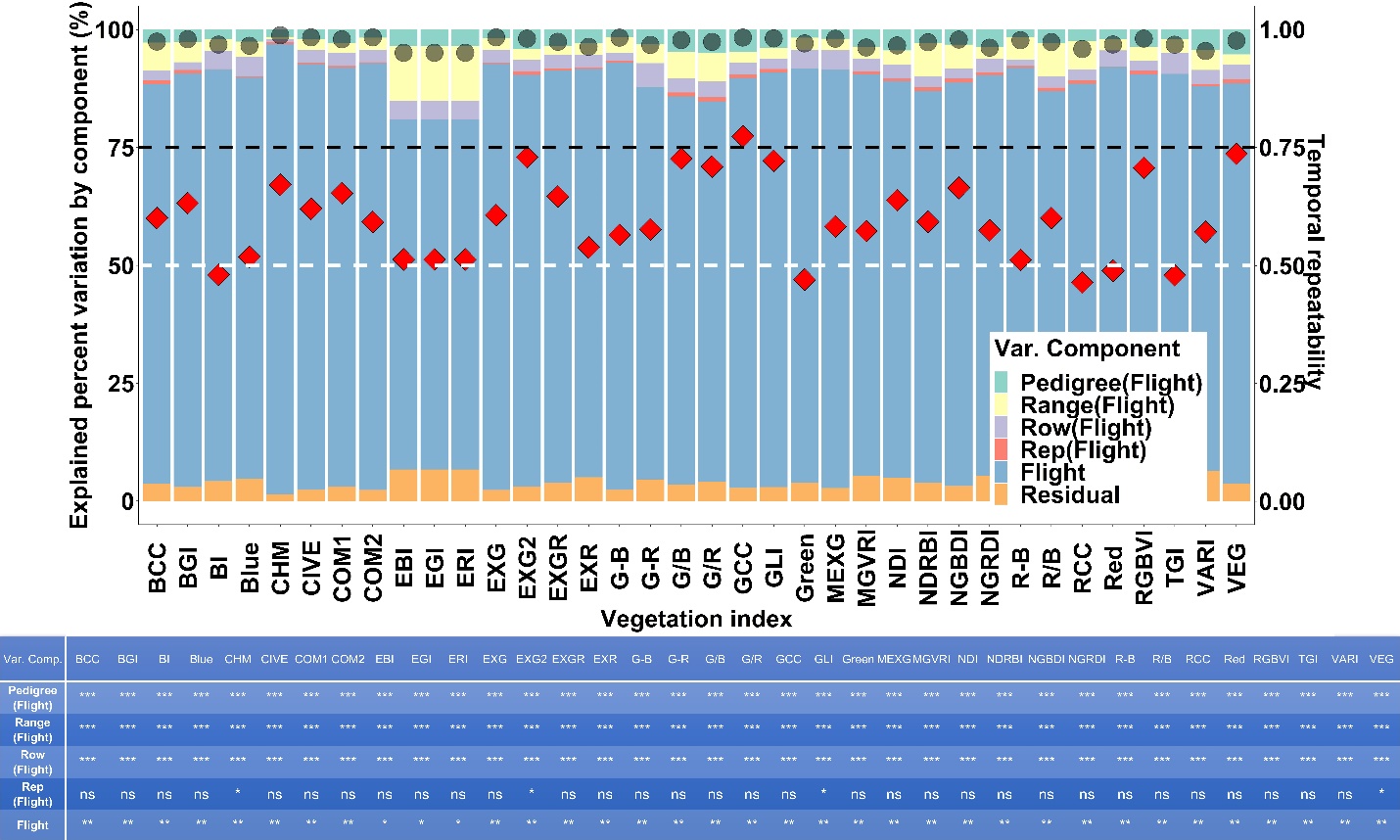


**Figure S2** stacked bar plots show the explained percent variation by each component in *Eq. 2* for each temporal trait in TPP_RGB. Left y axis corresponds to the explained percent variation of the components in the stacked bar plots while the right y axis shows the temporal repeatability (red diamonds calculated by Eq 3) and R^2^ values (black round symbols). Gray and black horizontal dashed lines represent the values of 0.50 and 0.75 where most of the temporal repeatability values of temporal traits accumulated. The table below shows the significance values of each component in *Eq. 2* for each temporal trait; ***, **, * are the 0.001, 0.01 and 0.05 significance levels respectively while ns is not statistically significant.


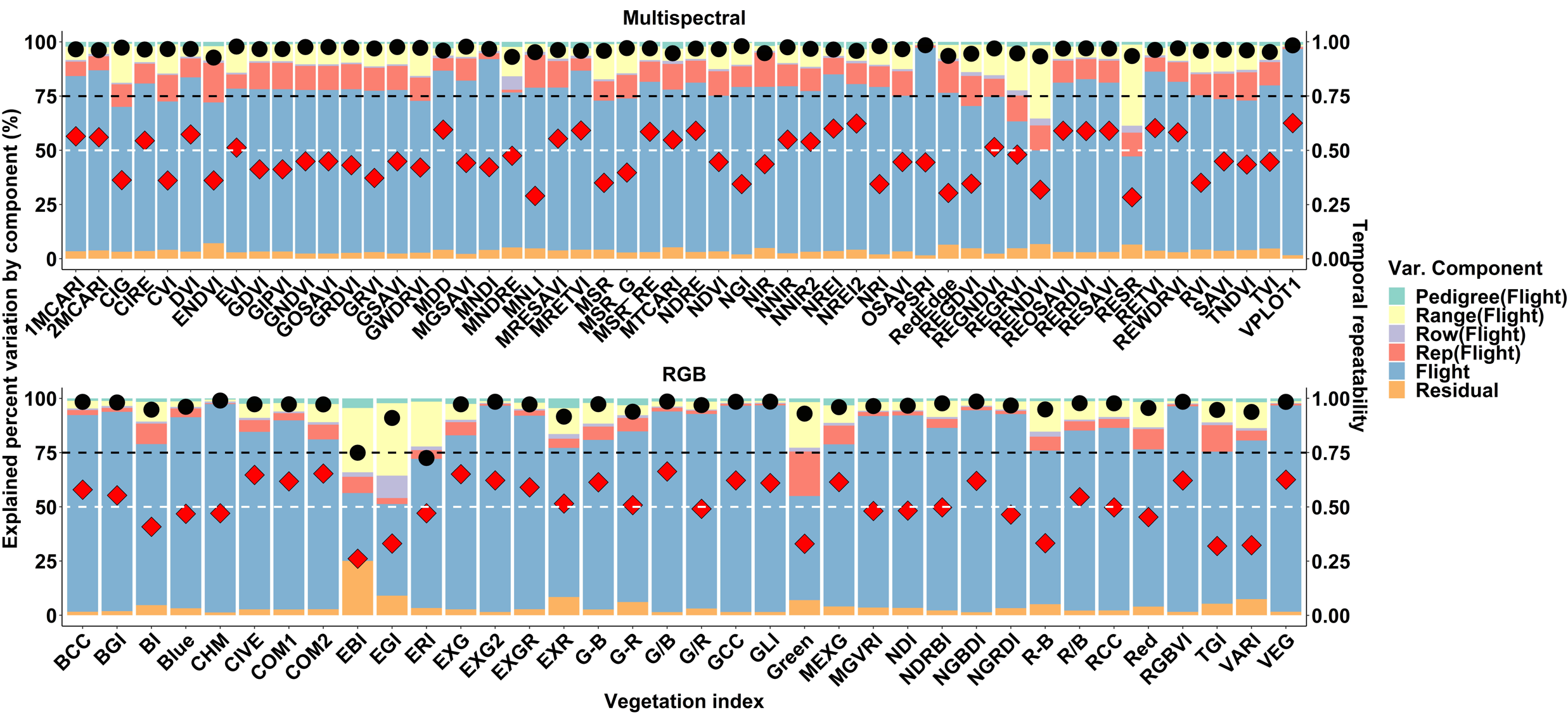


**Figure S3** stacked bar plots represent the explained percent variation by each component in *Eq. 2* for each temporal trait in TPP_Multi. Left y axis corresponds to the explained percent variation of the components while right y axis shows the temporal repeatability (red diamonds calculated by *Eq. 3*) and R^2^ values (black round symbols). Gray and black horizontal dashed lines represent the values of 0.50 and 0.75 where most of the temporal repeatability values of temporal traits accumulated.


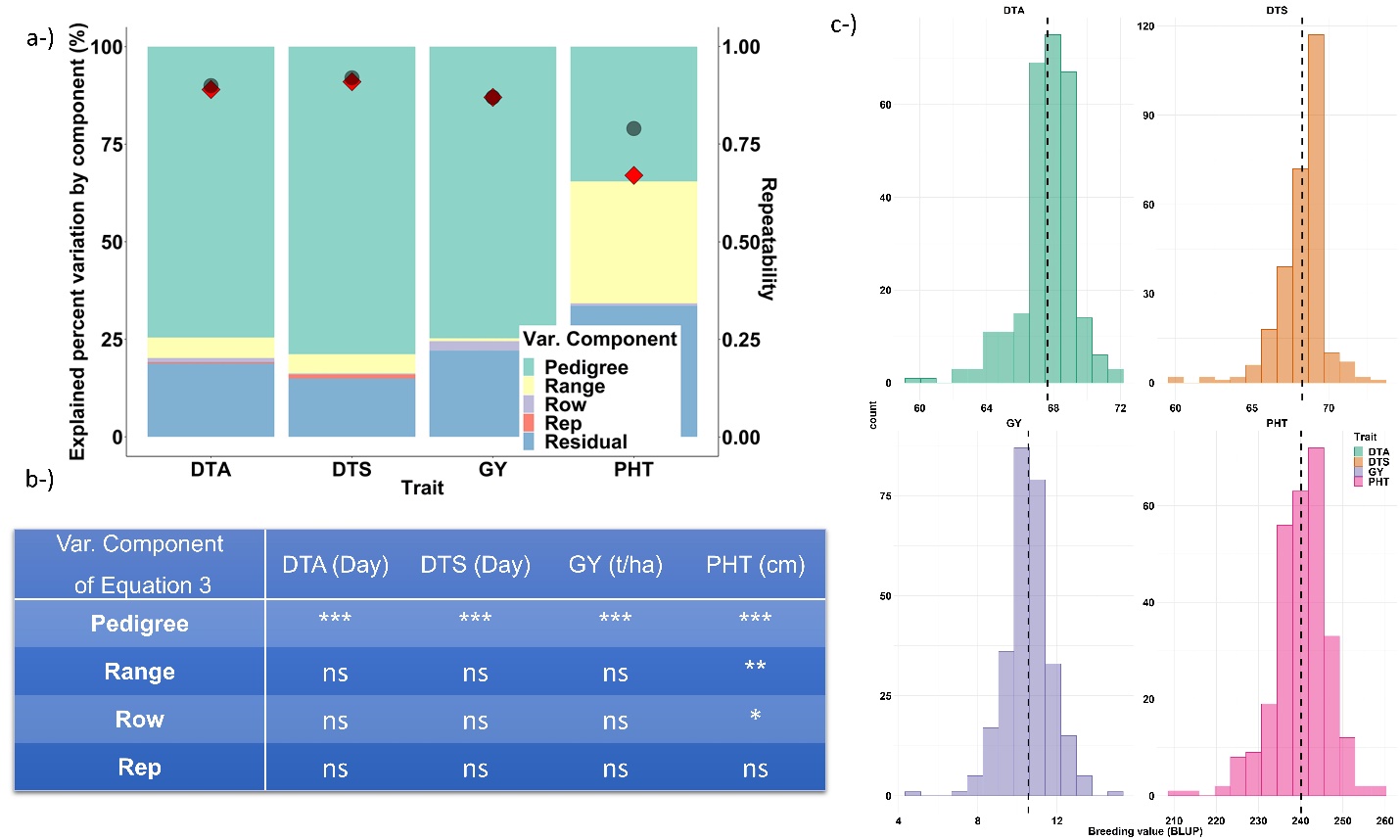


**Figure S4** a-) stacked bar plots shows the explained percent variation by each component in *Eq. 4* for each agronomic trait of days to anthesis (DTA), days to silking (DTS), grain yield (GY), and plant height (PHT). Left y axis represents the explained percent variation of the components while the right y axis shows the temporal repeatability (red diamonds calculated by *Eq. 5*) and Rsquared values (black round symbols). b-) The table shows the significance values of each component in *Eq. 4* for each temporal trait; ***, **, * are the 0.001, 0.01 and 0.05 significance levels respectively while ns is not statistically significant. c-) shows the histograms of the breeding values of each trait with their means represented by vertical black lines.


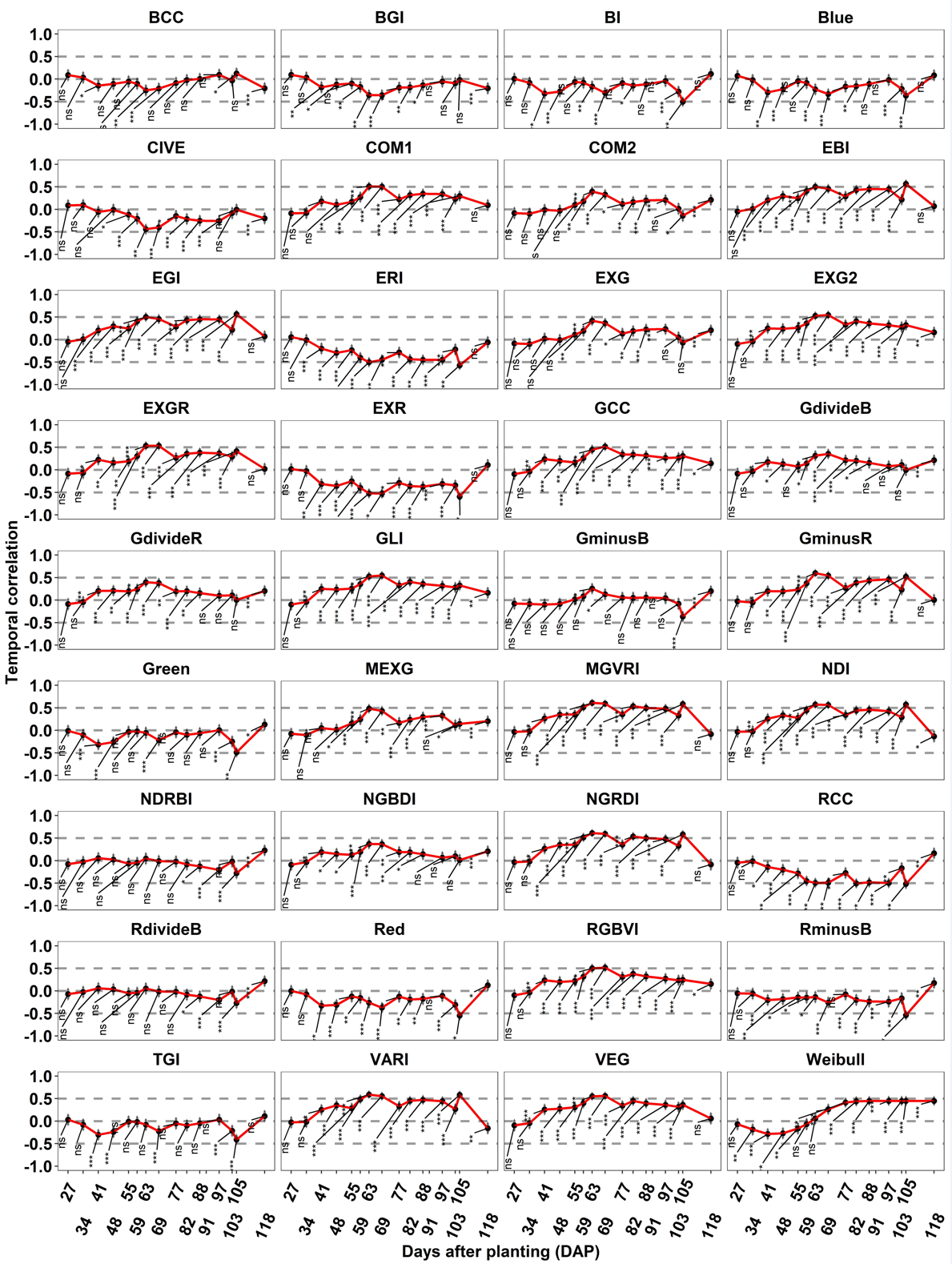


**Figure S5** Pearson correlation coefficients between the image derived temporal phenotypic values and grain yield (GY) at each flight time point of the VIs in TPP_RGB. Whiskers shows upper and lower confident intervals of temporal correlation based on 95 percent confidence level. Above, middle, and below dashed lines represent the 0.5, 0 and -0.5 temporal correlation values. ***, **, * are the 0.001, 0.01 and 0.05 significance levels respectively while ns is not statistically significant.


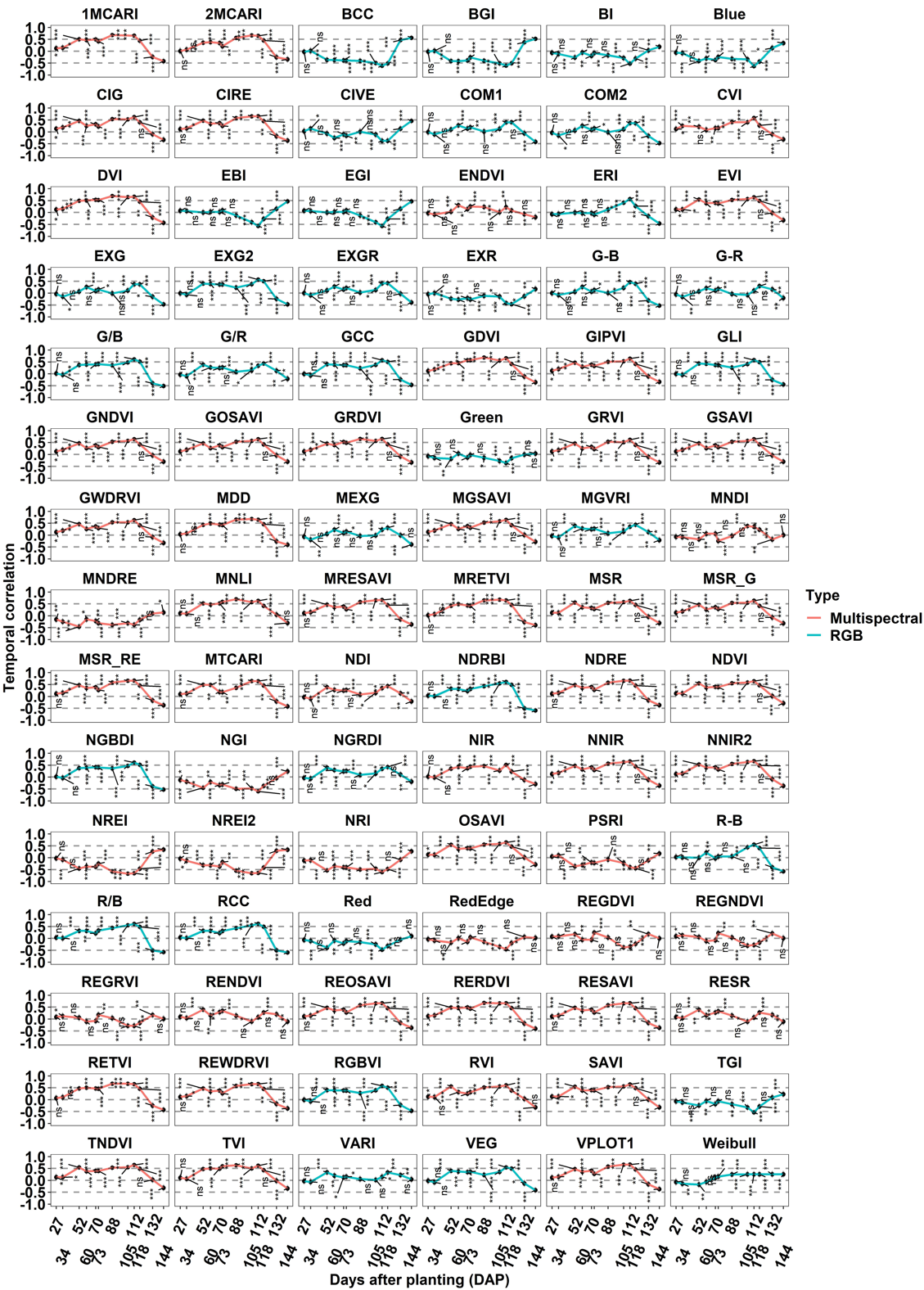


**Figure S6** Pearson correlation between the image derived temporal phenotype values and grain yield (GY) at each flight time point of the VIs in TPP_Multi. Each title separates vegetation indices according to their derivation from multispectral bands and RGB bands belonging to multispectral camera. Whiskers show upper and lower confidence intervals of temporal correlation based on 95 percent confidence levels. Above, middle, and below dashed lines represent the 0.5, 0 and -0.5 temporal correlation values. ***, **, * are the 0.001, 0.01 and 0.05 significance levels respectively; ns is not statistically significant.


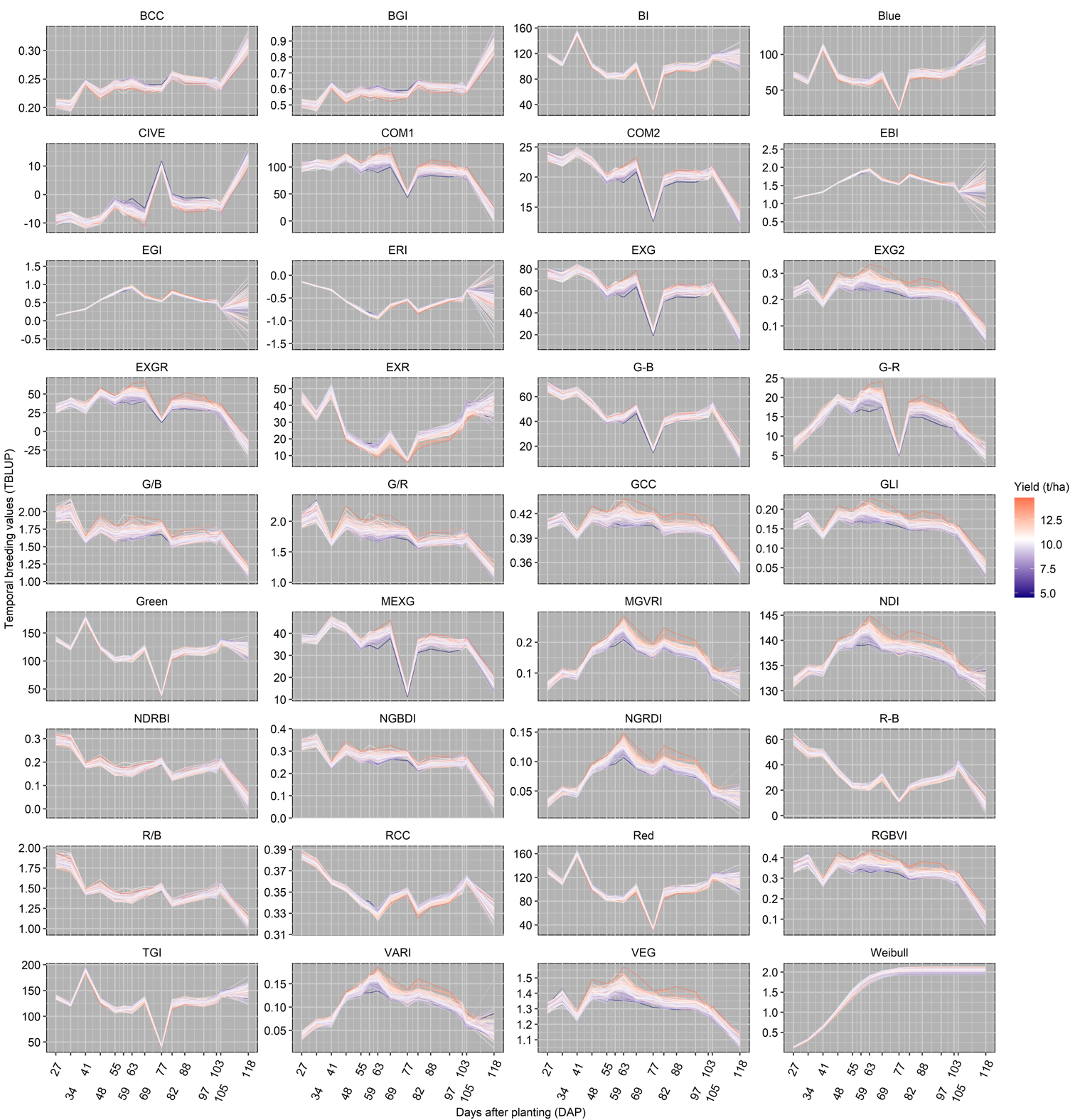


**Figure S7** temporal genotypic values for each of 280 maize hybrids predicted by *Eq. 2* belonging to each vegetation indices and Weibull_CHM in TPP_RGB of optimal management. Each hybrid was colored according to their yield values that are low, average, and high yielding represented by blue, white, and red respectively in the heatmap scale. Average yield was 10.5 t/ha.


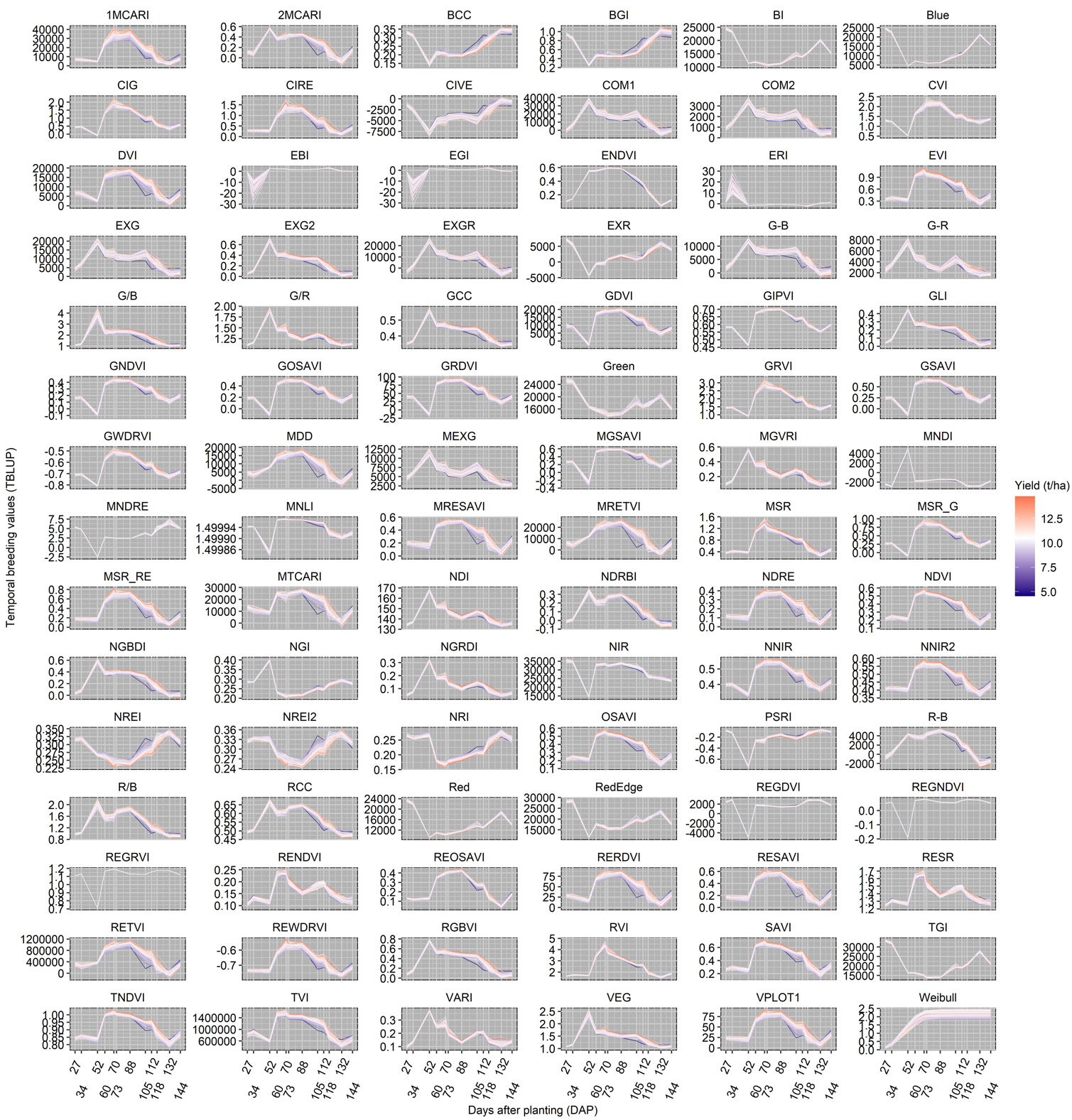


**Figure S8** temporal genotypic values for each of 280 maize hybrids predicted by *Eq. 2* belonging to each vegetation indices in TPP_Multi of optimal management. Each hybrid was colored according to their yield values that are low, average, and high yielding represented by blue, white, and red respectively in the heatmap scale. Average yield was 10.5 t/ha.


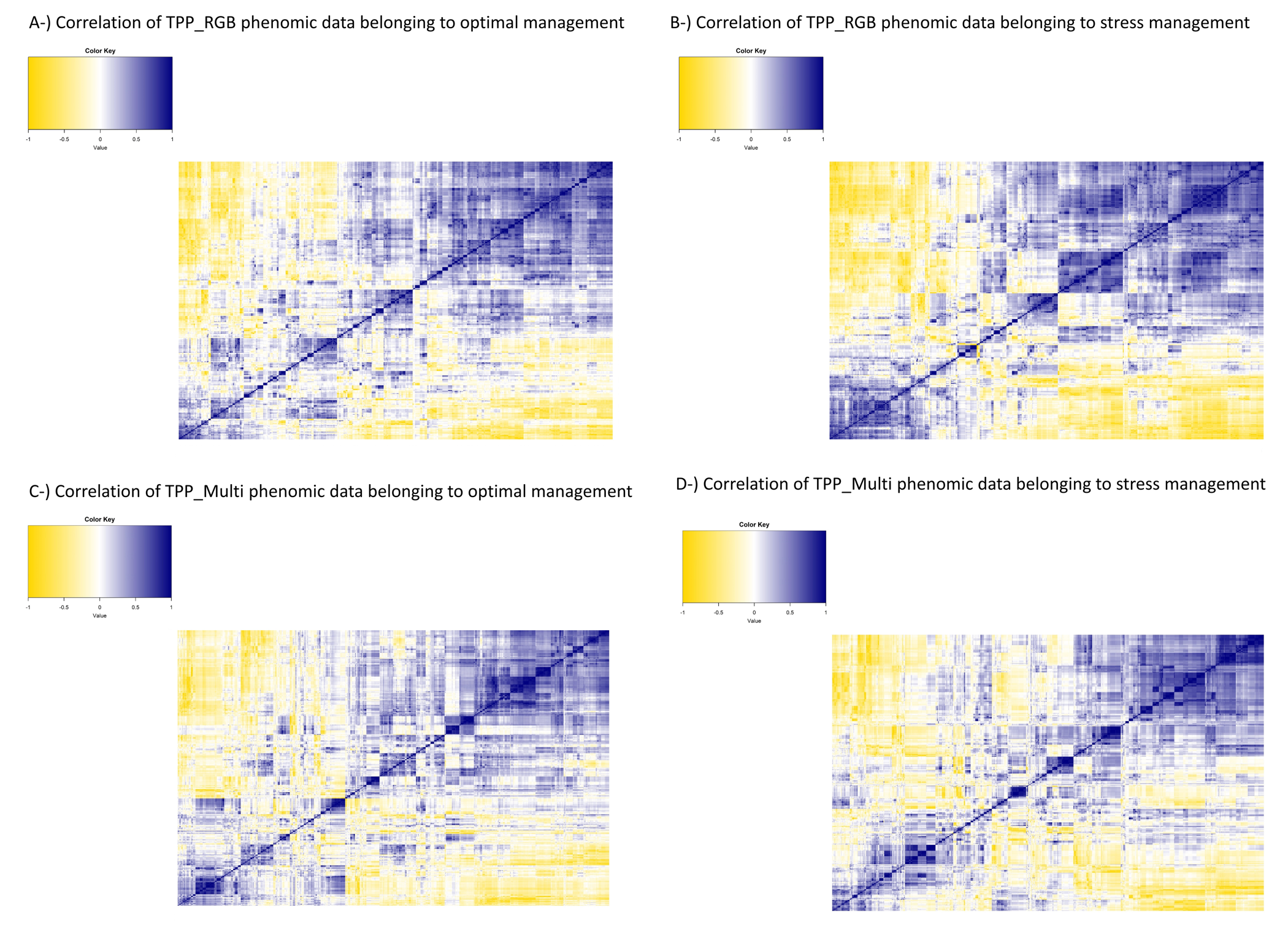


**Figure S9** Heatmap illustration of correlation matrix of each phenomic data used in phenomic prediction. A-) correlation of the phenomic data of optimal management derived from the low altitude high resolution rotary RGB (red-green-blue) platform. B-) Correlation of the phenomic data of stress management derived from RGB platform. C-) Correlation of the phenomic data of optimal management derived from the high altitude lower resolution multispectral HTP platform. D-) Correlation of the phenomic data of stress management derived from multispectral HTP platform.


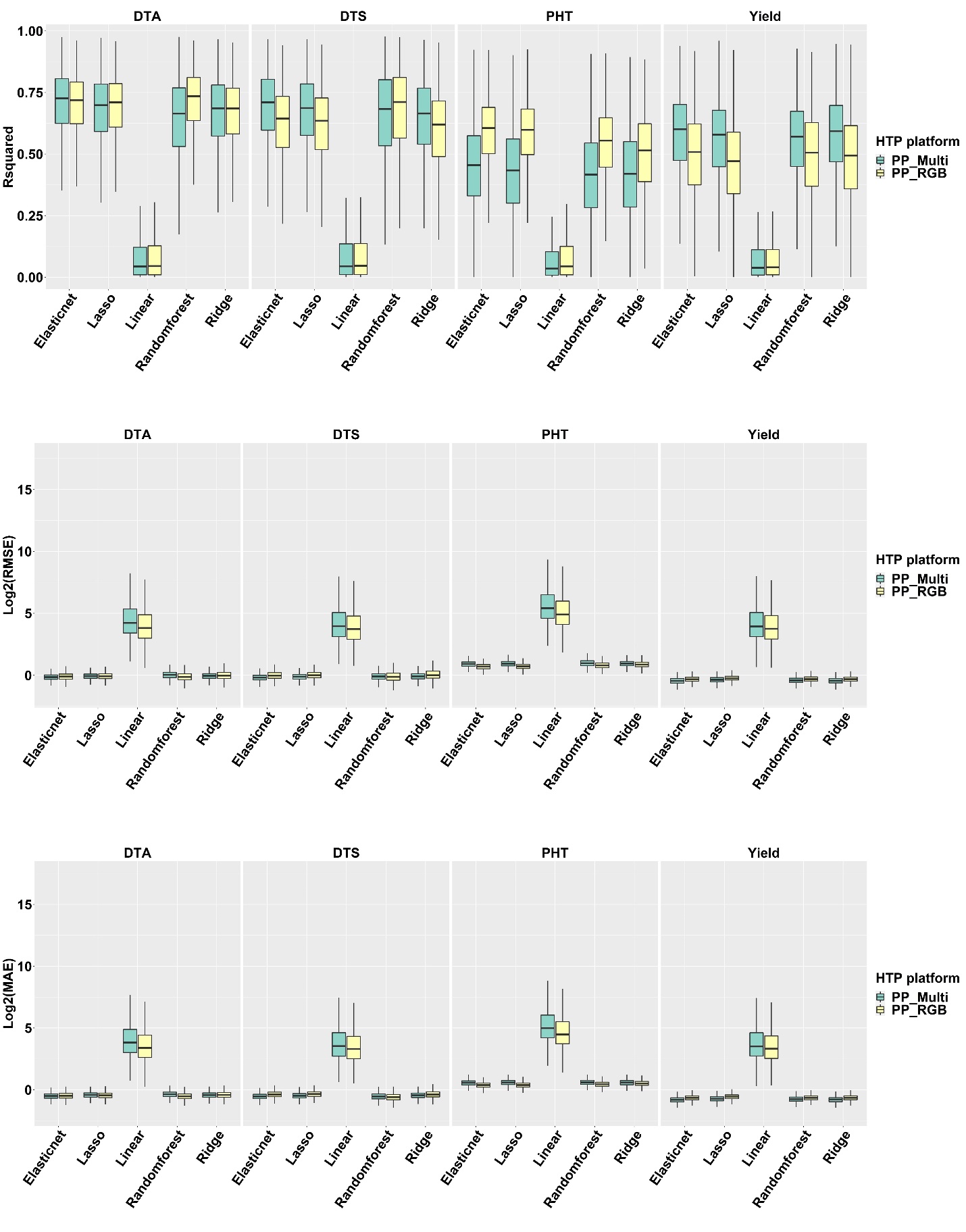


**Figure S10** R-squared, root mean square error (RMSE), and mean absolute error (MAE) values from top to bottom belonging to each model (on the x axis) and each predicted variable (from left to right). Log_2_() transformation was applied to RMSE and MAE values to show the excessive values belonging to linear model.


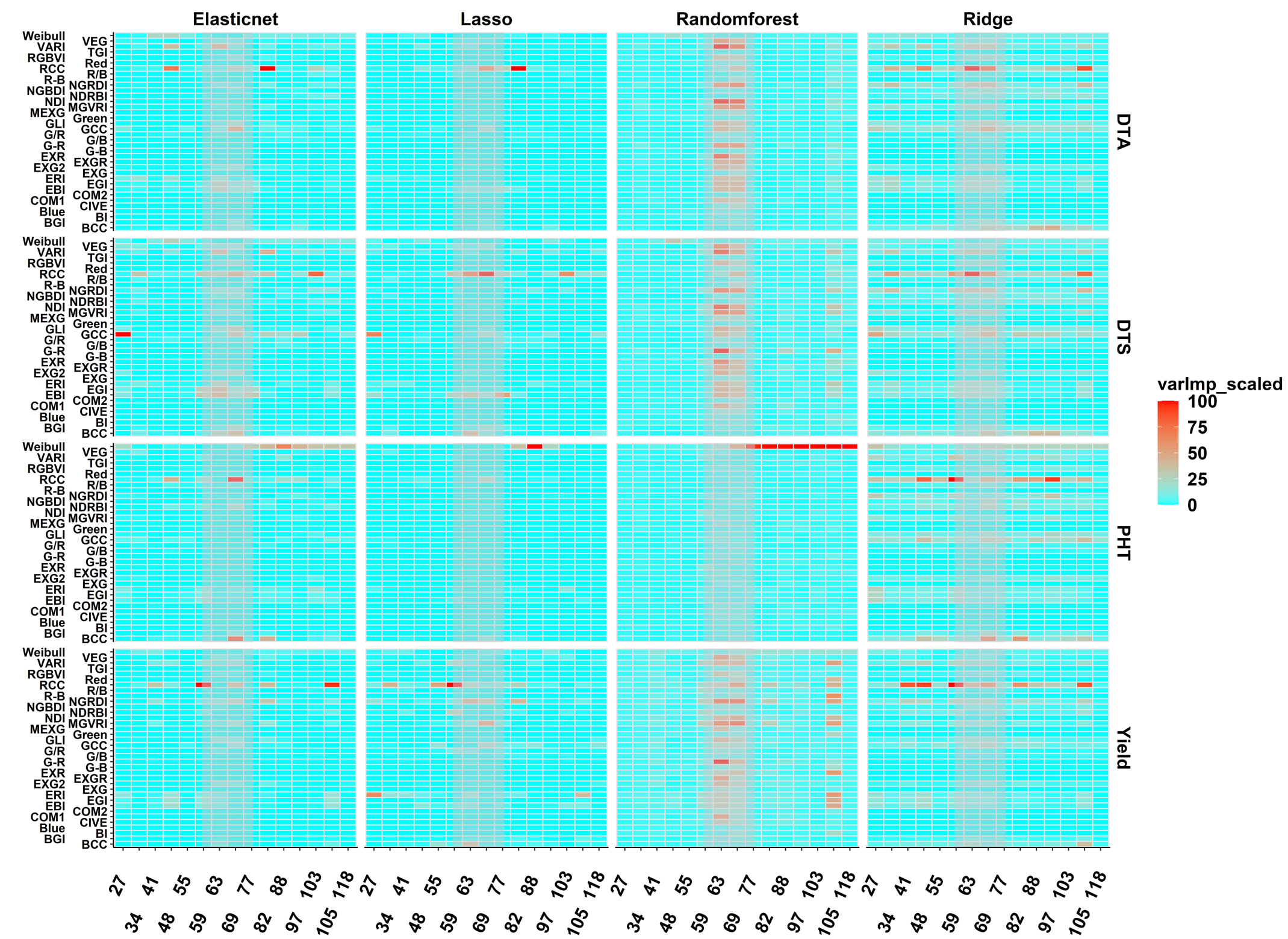


**Figure S11** Variable importance scores belonging to each predicted variable (from top to bottom) and model (from left to right) when TPP_RGB phenomic data was used. Y axis shows the VIs as well as Weibull_CHM and X axis shows the flight dates as days after planting times. The highlighted grey columns correspond to the range of flowering dates in this population.


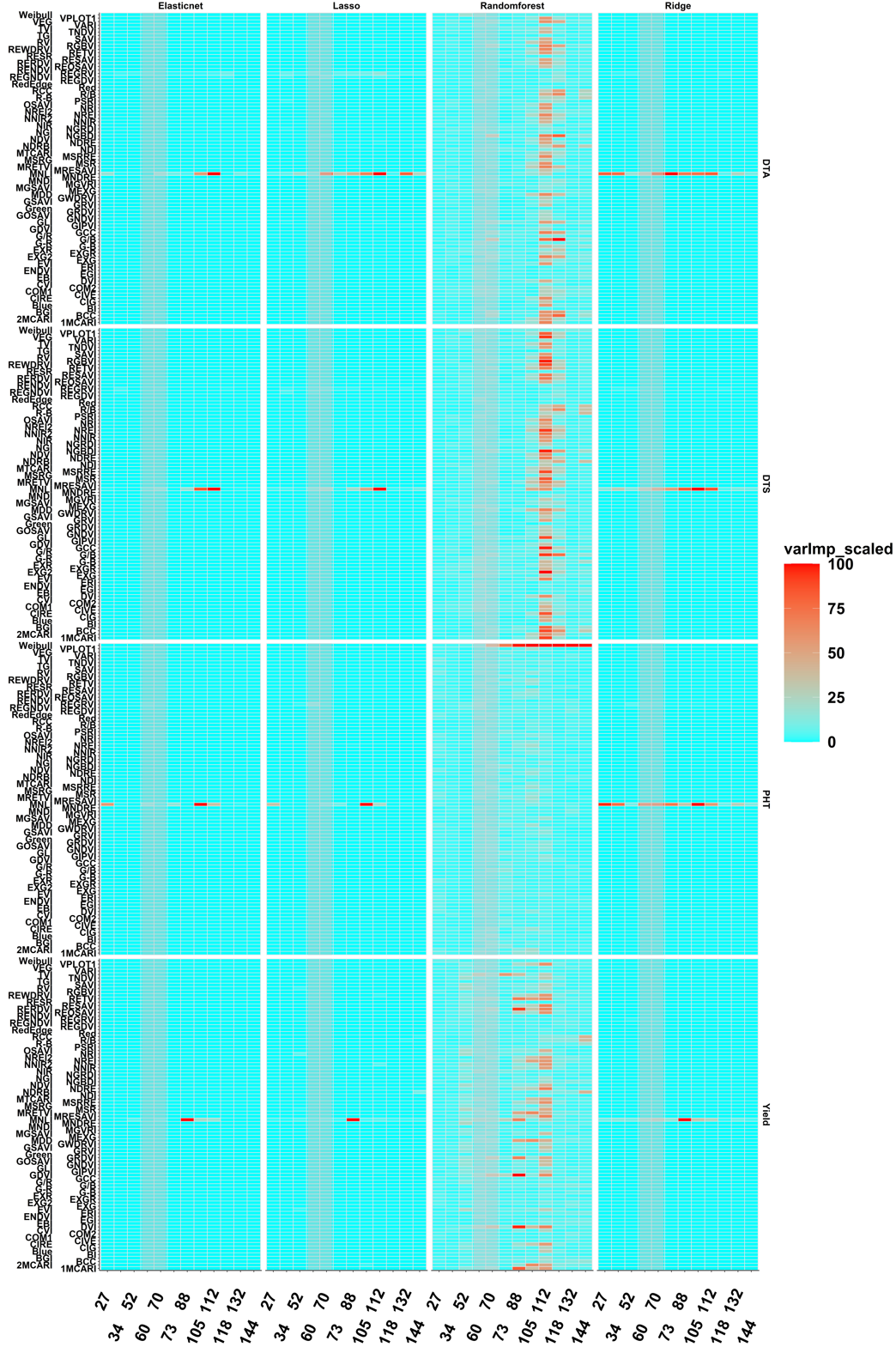


**Figure S12** Variable importance scores belonging to each predicted variable (from top to bottom) and model (from left to right) when TPP_Multi phenomic data was used. Y axis shows the VIs as well as Weibull _CHM and X axis shows the flight dates as days after planting times. The highlighted grey columns correspond to the range of flowering dates in this population.


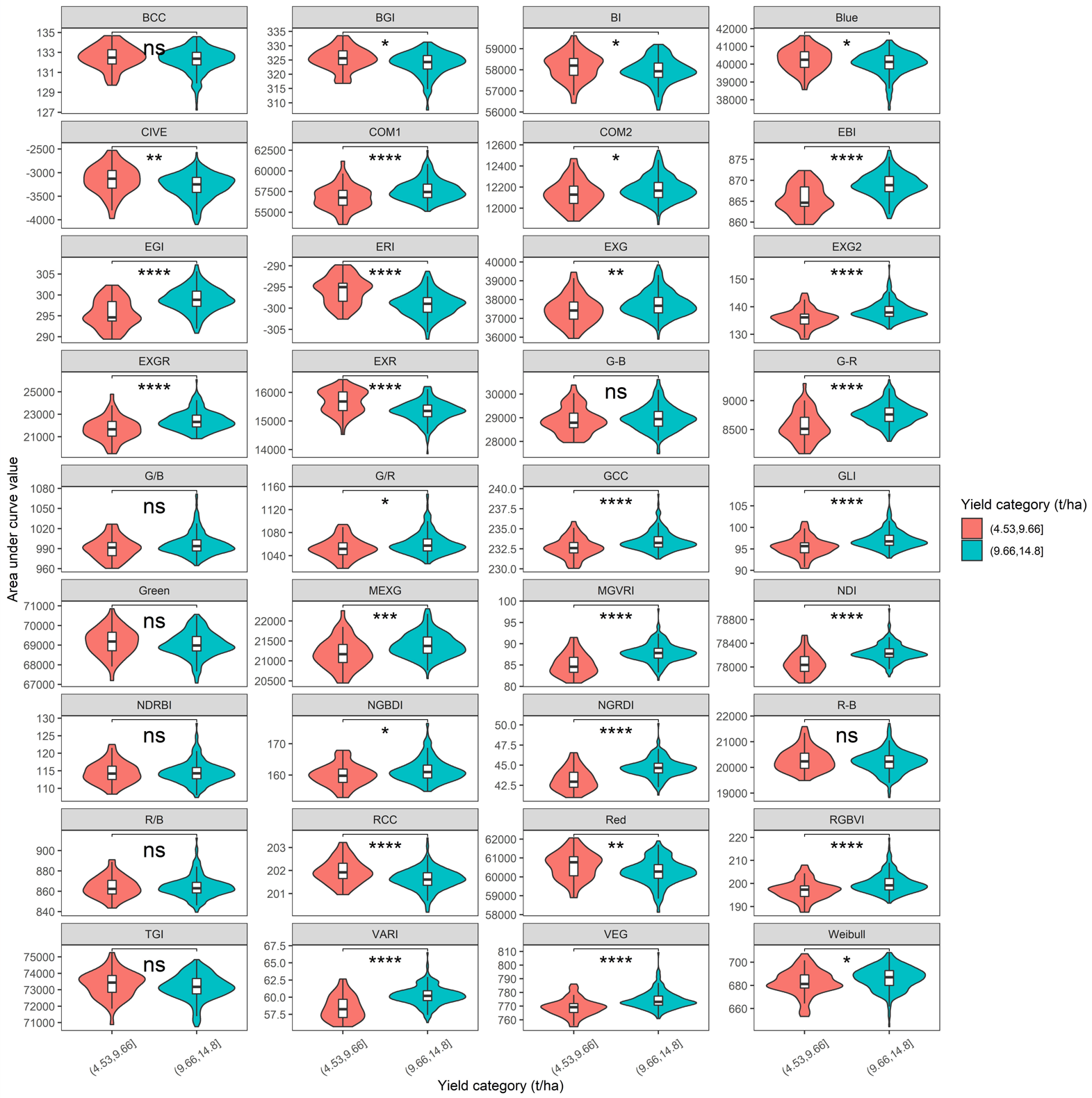


**Figure S13** Violin plots represent the area under curve value of each VI as well as Weibull _CHM for each genotype calculated by *Eq. 6* in TPP_RGB phenomic data belonging to optimal management. The area under curve values were divided into two equal bin categories based on genotype yield value as shown in the legend and each violin plot was colored based on these yield bins. The mean of each yield bin category was compared for each vegetation index using the Kruskal Wallis test to discover the VIs that can separate the low and high yielding bin categories. ns: p > 0.05, *: p <= 0.05, **: p <= 0.01, ***: p <= 0.001, ****: p <= 0.0001.


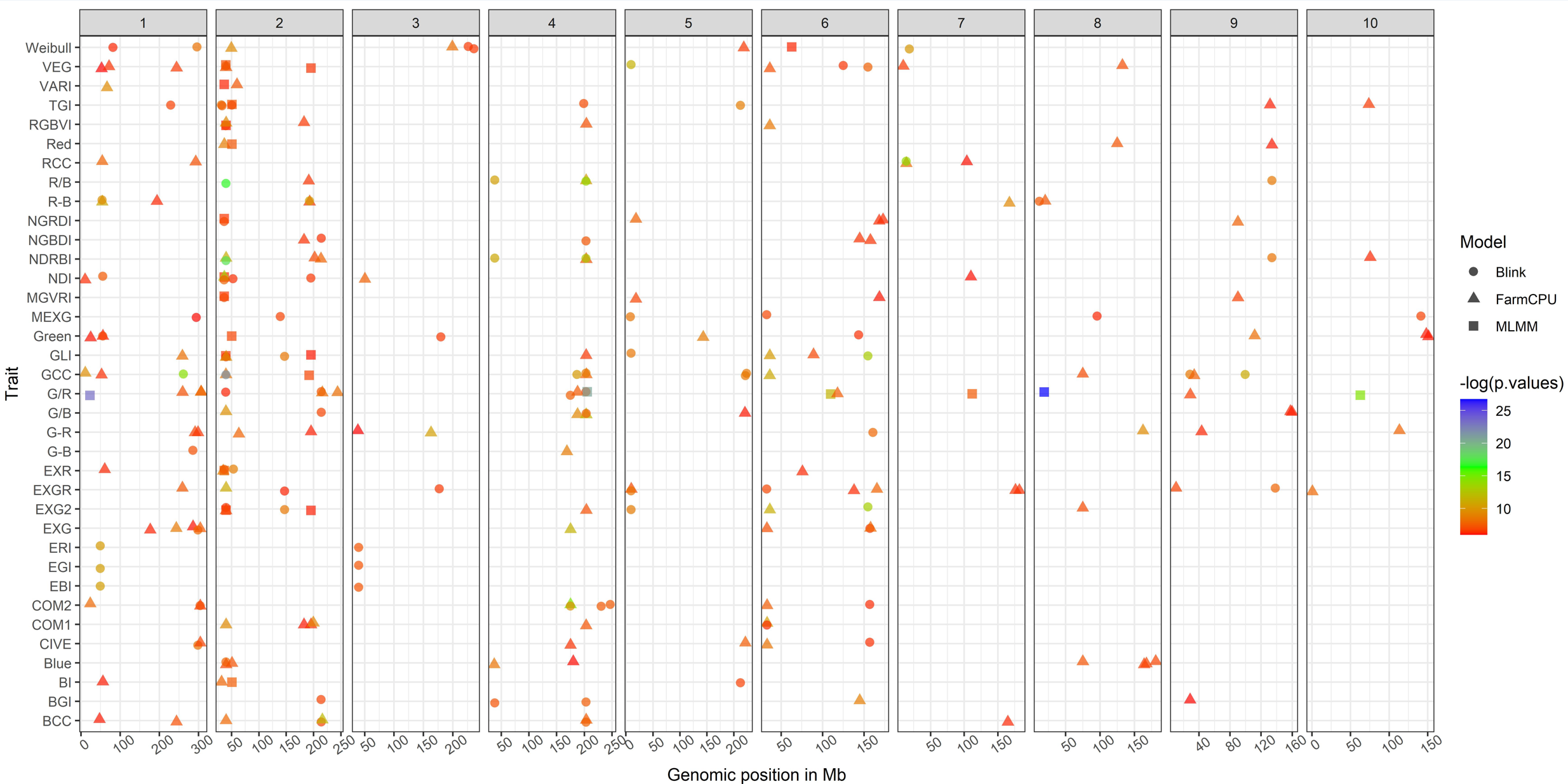


**Figure S14** Three GWAS models were run for all VIs and Weibull _CHM in TPP_RGB. Combined Manhattan plots results for all VI and Weibull _CHM (on the y-axis) in TPP_RGB were given. Below x-axis shows the genomic position of each chromosome. The heatmap legend shows the probability (-log10) of GWAS peaks between 6 to 26. Round, triangle and squares represent the Blink, FarmCPU and MLMM GWAS models results respectively.


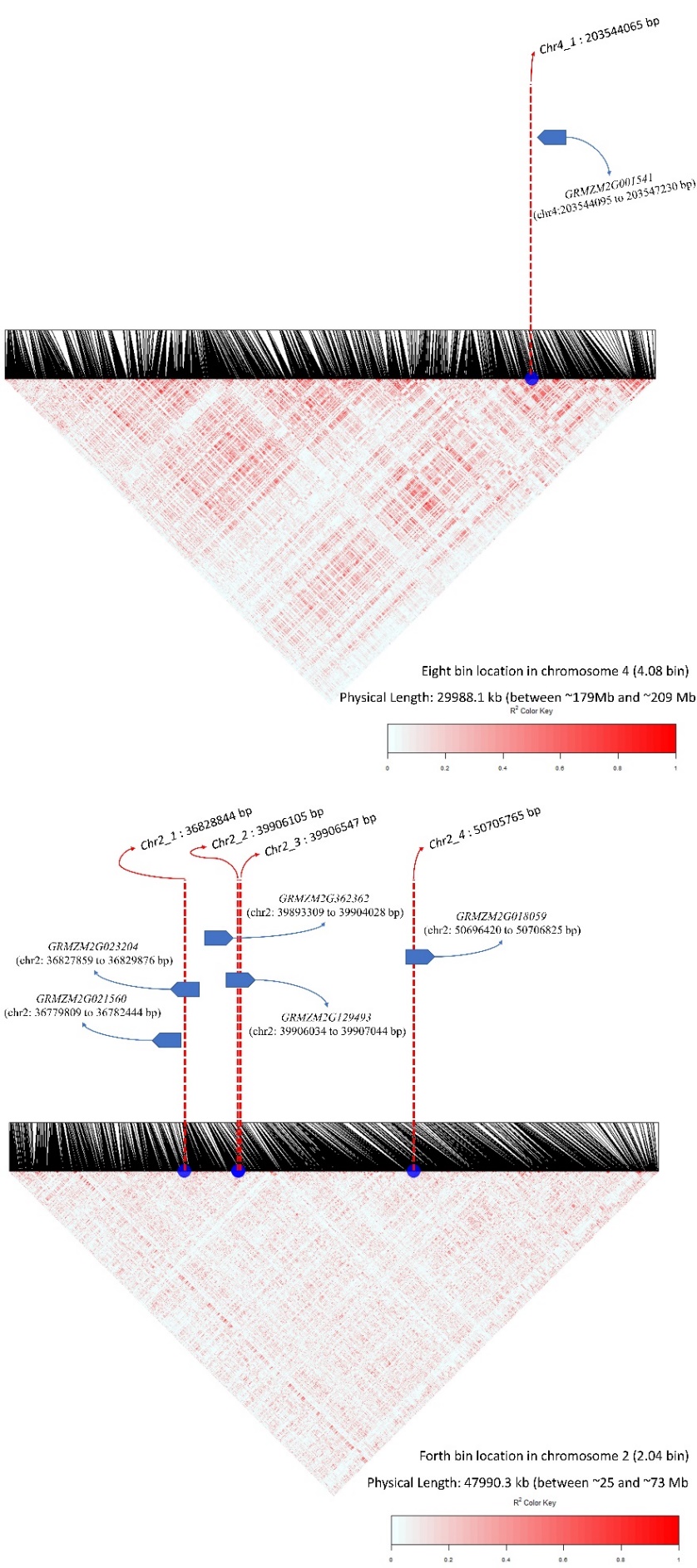


**Figure S15** LD blocks around Chromosome two loci, *chr2_1, chr2_2, chr2_3, chr2_4* (below) and *chr4_1* (above) loci, nearby LD blocks of these loci within the 2.04 and 4.08 genomic bin locations and candidate gene annotations.

**SI References**

Barnes, E., T. Clarke, S. Richards, P. Colaizzi, J. Haberland *et al.*, 2000 Coincident detection of crop water stress, nitrogen status and canopy density using ground based multispectral data in *Proceedings of the Fifth International Conference on Precision Agriculture, Bloomington, MN, USA*.

Bates, D., M. Mächler, B. Bolker, and S. Walker, 2014 Fitting linear mixed-effects models using lme4. *arXiv preprint arXiv:1406.5823*.

Bendig, J., K. Yu, H. Aasen, A. Bolten, S. Bennertz *et al.*, 2015 Combining UAV-based plant height from crop surface models, visible, and near infrared vegetation indices for biomass monitoring in barley. *International Journal of Applied Earth Observation and Geoinformation* 39:79-87.

Bradbury, P.J., Z. Zhang, D.E. Kroon, T.M. Casstevens, Y. Ramdoss *et al.*, 2007 TASSEL: software for association mapping of complex traits in diverse samples. *Bioinformatics* 23 (19):2633-2635.

Broge, N.H., and E. Leblanc, 2001 Comparing prediction power and stability of broadband and hyperspectral vegetation indices for estimation of green leaf area index and canopy chlorophyll density. *Remote sensing of environment* 76 (2):156-172.

Burgos-Artizzu, X.P., A. Ribeiro, M. Guijarro, and G. Pajares, 2011 Real-time image processing for crop/weed discrimination in maize fields. *Computers and Electronics in Agriculture* 75 (2):337-346.

Buschmann, C., and E. Nagel, 1993 In vivo spectroscopy and internal optics of leaves as basis for remote sensing of vegetation. *International Journal of Remote Sensing* 14 (4):711-722.

Cao, Q., Y. Miao, H. Wang, S. Huang, S. Cheng *et al.*, 2013 Non-destructive estimation of rice plant nitrogen status with Crop Circle multispectral active canopy sensor. *Field Crops Research* 154:133-144.

Chen, J.M., 1996 Evaluation of vegetation indices and a modified simple ratio for boreal applications. *Canadian Journal of Remote Sensing* 22 (3):229-242.

Crippen, R.E., 1990 Calculating the vegetation index faster. *Remote sensing of Environment* 34 (1):71-73.

Datt, B., 1999 Visible/near infrared reflectance and chlorophyll content in Eucalyptus leaves. *International Journal of Remote Sensing* 20 (14):2741-2759.

Daughtry, C.S., C. Walthall, M. Kim, E.B. De Colstoun, and J. McMurtrey Iii, 2000 Estimating corn leaf chlorophyll concentration from leaf and canopy reflectance. *Remote sensing of Environment* 74 (2):229-239.

Elsayed, S., P. Rischbeck, and U. Schmidhalter, 2015 Comparing the performance of active and passive reflectance sensors to assess the normalized relative canopy temperature and grain yield of drought-stressed barley cultivars. *Field Crops Research* 177:148-160.

Elshire, R.J., J.C. Glaubitz, Q. Sun, J.A. Poland, K. Kawamoto *et al.*, 2011 A robust, simple genotyping-by-sequencing (GBS) approach for high diversity species. *PloS one* 6 (5):e19379.

Endelman, J.B., 2011 Ridge regression and other kernels for genomic selection with R package rrBLUP. *The plant genome* 4 (3).

Erdle, K., B. Mistele, and U. Schmidhalter, 2011 Comparison of active and passive spectral sensors in discriminating biomass parameters and nitrogen status in wheat cultivars. *Field Crops Research* 124 (1):74-84.

Escadafal, R., 1993 Remote sensing of soil color: principles and applications. *Remote Sensing Reviews* 7 (3-4):261-279.

Ferrari, S., D. Vairo, F.M. Ausubel, F. Cervone, and G. De Lorenzo, 2003 Tandemly duplicated Arabidopsis genes that encode polygalacturonase-inhibiting proteins are regulated coordinately by different signal transduction pathways in response to fungal infection. *The Plant Cell* 15 (1):93-106.

Gitelson, A.A., 2004 Wide dynamic range vegetation index for remote quantification of biophysical characteristics of vegetation. *Journal of plant physiology* 161 (2):165-173.

Gitelson, A.A., Y.J. Kaufman, and M.N. Merzlyak, 1996 Use of a green channel in remote sensing of global vegetation from EOS-MODIS. *Remote sensing of Environment* 58 (3):289-298.

Gitelson, A.A., Y.J. Kaufman, R. Stark, and D. Rundquist, 2002 Novel algorithms for remote estimation of vegetation fraction. *Remote sensing of Environment* 80 (1):76-87.

Gitelson, A.A., A. Vina, V. Ciganda, D.C. Rundquist, and T.J. Arkebauer, 2005 Remote estimation of canopy chlorophyll content in crops. *Geophysical Research Letters* 32 (8).

Glaubitz, J.C., T.M. Casstevens, F. Lu, J. Harriman, R.J. Elshire *et al.*, 2014 TASSEL-GBS: a high capacity genotyping by sequencing analysis pipeline. *PloS one* 9 (2):e90346.

Golzarian, M.R., and R.A. Frick, 2011 Classification of images of wheat, ryegrass and brome grass species at early growth stages using principal component analysis. *Plant Methods* 7 (1):1-11.

Gong, P., R. Pu, G.S. Biging, and M.R. Larrieu, 2003 Estimation of forest leaf area index using vegetation indices derived from Hyperion hyperspectral data. *IEEE transactions on geoscience and remote sensing* 41 (6):1355-1362.

Guerrero, J.M., G. Pajares, M. Montalvo, J. Romeo, and M. Guijarro, 2012 Support vector machines for crop/weeds identification in maize fields. *Expert Systems with Applications* 39 (12):11149-11155.

Guijarro, M., G. Pajares, I. Riomoros, P. Herrera, X. Burgos-Artizzu *et al.*, 2011 Automatic segmentation of relevant textures in agricultural images. *Computers and Electronics in Agriculture* 75 (1):75-83.

Haboudane, D., J.R. Miller, E. Pattey, P.J. Zarco-Tejada, and I.B. Strachan, 2004 Hyperspectral vegetation indices and novel algorithms for predicting green LAI of crop canopies: Modeling and validation in the context of precision agriculture. *Remote sensing of environment* 90 (3):337-352.

Haboudane, D., J.R. Miller, N. Tremblay, P.J. Zarco-Tejada, and L. Dextraze, 2002 Integrated narrow-band vegetation indices for prediction of crop chlorophyll content for application to precision agriculture. *Remote sensing of environment* 81 (2-3):416-426.

Hague, T., N. Tillett, and H. Wheeler, 2006 Automated crop and weed monitoring in widely spaced cereals. *Precision Agriculture* 7 (1):21-32.

Hattan, J., H. Kanamoto, M. Takemura, A. Yokota, and T. Kohchi, 2004 Molecular characterization of the cytoplasmic interacting protein of the receptor kinase IRK expressed in the inflorescence and root apices of Arabidopsis. *Bioscience, biotechnology, and biochemistry* 68 (12):2598-2606.

He, C., Y. Du, J. Fu, E. Zeng, S. Park *et al.*, 2020 Early drought-responsive genes are variable and relevant to drought tolerance. *G3: Genes, Genomes, Genetics* 10 (5):1657-1670.

Huete, A., K. Didan, T. Miura, E.P. Rodriguez, X. Gao *et al.*, 2002 Overview of the radiometric and biophysical performance of the MODIS vegetation indices. *Remote sensing of environment* 83 (1-2):195-213.

Huete, A.R., 1988 A soil-adjusted vegetation index (SAVI). *Remote sensing of environment* 25 (3):295-309.

Hunt, E.R., M. Cavigelli, C.S. Daughtry, J.E. Mcmurtrey, and C.L. Walthall, 2005 Evaluation of digital photography from model aircraft for remote sensing of crop biomass and nitrogen status. *Precision Agriculture* 6 (4):359-378.

Hunt, E.R., C. Daughtry, J.U. Eitel, and D.S. Long, 2011 Remote sensing leaf chlorophyll content using a visible band index.

Jasper, J., S. Reusch, and A. Link, 2009 Active sensing of the N status of wheat using optimized wavelength combination: impact of seed rate, variety and growth stage. *Precision agriculture* 9:23-30.

Jordan, C.F., 1969 Derivation of leaf‐area index from quality of light on the forest floor. *Ecology* 50 (4):663-666.

Kataoka, T., T. Kaneko, H. Okamoto, and S. Hata, 2003 Crop growth estimation system using machine vision, pp. b1079-b1083 vol. 1072 in *Proceedings 2003 IEEE/ASME International Conference on Advanced Intelligent Mechatronics (AIM 2003)*. IEEE.

Kraus, K., and N. Pfeifer, 1998 Determination of terrain models in wooded areas with airborne laser scanner data. *ISPRS Journal of Photogrammetry and remote Sensing* 53 (4):193-203.

Le Maire, G., C. Francois, and E. Dufrene, 2004 Towards universal broad leaf chlorophyll indices using PROSPECT simulated database and hyperspectral reflectance measurements. *Remote sensing of environment* 89 (1):1-28.

Liu, L., Y. Du, X. Shen, M. Li, W. Sun *et al.*, 2015 KRN4 controls quantitative variation in maize kernel row number. *PLoS genetics* 11 (11):e1005670.

Louhaichi, M., M.M. Borman, and D.E. Johnson, 2001 Spatially located platform and aerial photography for documentation of grazing impacts on wheat. *Geocarto International* 16 (1):65-70.

McFarland, B.A., N. AlKhalifah, M. Bohn, J. Bubert, E.S. Buckler *et al.*, 2020 Maize genomes to fields (G2F): 2014–2017 field seasons: genotype, phenotype, climatic, soil, and inbred ear image datasets. *BMC research notes* 13 (1):1-6.

Merzlyak, M.N., A.A. Gitelson, O.B. Chivkunova, and V.Y. Rakitin, 1999 Non‐destructive optical detection of pigment changes during leaf senescence and fruit ripening. *Physiologia plantarum* 106 (1):135-141.

Meyer, G., T. Hindman, and K. Laksmi, 1998 MG (ed.), Deshazer JA, Machine vision detection parameters for plant species identification. *Precision agri540 culture and biological quality, Boston, Massachusetts, USA* 3 (4):3543.

Meyer, G.E., and J.C. Neto, 2008 Verification of color vegetation indices for automated crop imaging applications. *Computers and electronics in agriculture* 63 (2):282-293.

Minic, Z., 2008 Physiological roles of plant glycoside hydrolases. *Planta* 227 (4):723-740.

Money, D., K. Gardner, Z. Migicovsky, H. Schwaninger, G.-Y. Zhong *et al.*, 2015 LinkImpute: fast and accurate genotype imputation for nonmodel organisms. *G3: Genes, Genomes, Genetics* 5 (11):2383-2390.

Qi, J., A. Chehbouni, A.R. Huete, Y.H. Kerr, and S. Sorooshian, 1994 A modified soil adjusted vegetation index. *Remote sensing of environment* 48 (2):119-126.

Richardson, A.J., and C. Wiegand, 1977 Distinguishing vegetation from soil background information. *Photogrammetric engineering and remote sensing* 43 (12):1541-1552.

Rondeaux, G., M. Steven, and F. Baret, 1996 Optimization of soil-adjusted vegetation indices. *Remote sensing of environment* 55 (2):95-107.

Roujean, J.-L., and F.-M. Breon, 1995 Estimating PAR absorbed by vegetation from bidirectional reflectance measurements. *Remote sensing of Environment* 51 (3):375-384.

Sandham, L., and H. Zietsman, 1997 Surface temperature measurement from space: a case study in the south western cape of South Africa. *South African Journal of Enology and Viticulture* 18 (2):25-30.

Shin, J.-H., S. Blay, B. McNeney, and J. Graham, 2006 LDheatmap: an R function for graphical display of pairwise linkage disequilibria between single nucleotide polymorphisms. *Journal of statistical software* 16 (3):1-10.

Sripada, R.P., R.W. Heiniger, J.G. White, and A.D. Meijer, 2006 Aerial color infrared photography for determining early in‐season nitrogen requirements in corn. *Agronomy Journal* 98 (4):968-977.

Tucker, C.J., 1979 Red and photographic infrared linear combinations for monitoring vegetation. *Remote sensing of Environment* 8 (2):127-150.

Vincini, M., E. Frazzi, and P. D’Alessio, 2008 A broad-band leaf chlorophyll vegetation index at the canopy scale. *Precision Agriculture* 9 (5):303-319.

Wang, W., X. Yao, X. Yao, Y. Tian, X. Liu *et al.*, 2012 Estimating leaf nitrogen concentration with three-band vegetation indices in rice and wheat. *Field Crops Research* 129:90-98.

Wang, X., H. Wang, S. Liu, A. Ferjani, J. Li *et al.*, 2016 Genetic variation in ZmVPP1 contributes to drought tolerance in maize seedlings. *Nature genetics* 48 (10):1233-1241.

Wang, Y., Y. Wang, X. Wang, and D. Deng, 2020 Integrated meta-QTL and genome-wide association study analyses reveal candidate genes for maize yield. *Journal of Plant Growth Regulation* 39 (1):229-238.

Woebbecke, D.M., G.E. Meyer, K. Von Bargen, and D.A. Mortensen, 1995 Color indices for weed identification under various soil, residue, and lighting conditions. *Transactions of the ASAE* 38 (1):259-269.

Wu, X., Y. Li, Y. Shi, Y. Song, D. Zhang *et al.*, 2016 Joint‐linkage mapping and GWAS reveal extensive genetic loci that regulate male inflorescence size in maize. *Plant Biotechnology Journal* 14 (7):1551-1562.

Zarco-Tejada, P.J., A. Berjón, R. López-Lozano, J.R. Miller, P. Martín *et al.*, 2005 Assessing vineyard condition with hyperspectral indices: Leaf and canopy reflectance simulation in a row-structured discontinuous canopy. *Remote Sensing of Environment* 99 (3):271-287.

Zhou, Z., G. Li, S. Tan, D. Li, T.M. Weiß *et al.*, 2020 A QTL atlas for grain yield and its component traits in maize (Zea mays). *Plant Breeding* 139 (3):562-574.
